# Timing Evidence for Symbolic Phonological Representations and Phonology-Extrinsic Timing in Speech Production

**DOI:** 10.3389/fpsyg.2019.02952

**Published:** 2020-01-24

**Authors:** Alice Turk, Stefanie Shattuck-Hufnagel

**Affiliations:** ^1^Linguistics and English Language, School of Philosophy, Psychology and Language Sciences, The University of Edinburgh, Edinburgh, United Kingdom; ^2^Massachusetts Institute of Technology, Cambridge, MA, United States

**Keywords:** speech motor control, phonology, phonetics, symbolic phonological representations, timing

## Abstract

The goals of this paper are (1) to discuss the key features of existing articulatory models of speech production that govern their approaches to timing, along with advantages and disadvantages of each, and (2) to evaluate these features in terms of several pieces of evidence from both the speech and nonspeech motor control literature. This evidence includes greater timing precision at movement endpoints compared to other parts of movements, suggesting the separate control of the timing of movement endpoints compared to other parts of movement. This endpoint timing precision challenges models in which all parts of a movement trajectory are controlled by the same equation of motion, but supports models in which (a) abstract, symbolic phonological representations map onto spatial and temporal characteristics of the part(s) of movement most closely related to the goal of producing a planned set of acoustic cues to signal the phonological contrast (often the endpoint), (b) movements are coordinated primarily based on the goal-related part of movement, and (c) speakers give priority to the accurate implementation of the part(s) of movement most closely related to the phonological goals. In addition, this paper presents three types of evidence for phonology-extrinsic timing, suggesting that surface duration requirements are represented during speech production. Phonology-extrinsic timing is also supported by greater timing variability for repetitions of longer intervals, assumed to be due to noise in a general-purpose (and phonology-extrinsic) timekeeping process. The evidence appears to be incompatible with models that have a unified Phonology/Phonetics Component, that do not represent the surface timing of phonetic events, and do not represent, specify and track timing by general-purpose timekeeping mechanisms. Taken together, this evidence supports an alternative approach to modeling speech production that is based on symbolic phonological representations and general-purpose, phonology-extrinsic, timekeeping mechanisms, rather than on spatio-temporal phonological representations and phonology-specific timing mechanisms. Thus, the evidence suggests that models in that alternative framework should be developed, so they can be tested with the same rigor as have models based on spatio-temporal phonological representations with phonology-intrinsic timing.

## Introduction

There is growing appreciation that models of speech production need to take the process all the way to completion, i.e., to provide principled accounts of systematic patterns of timing behavior in speech, for individual movements and their coordination, and for intervals between acoustic landmarks (Stevens, 2002) that are created by these movements. The known timing characteristics of individual movements include their smooth, single-peaked velocity profiles, the strong positive relationship between peak velocity and distance (longer distance movements have higher peak velocities), and the increase in duration observed for more accurate and/or longer distance movements (in spite of higher peak velocities for longer distances), cf. Fitts’ (1954) law. Patterns of coordination between movements include the coordination of movements made by several articulators involved in creating a single constriction, as well as the coordination of overlapping movements involved in making sequences of constrictions. Timing patterns of intervals between acoustic landmarks include systematic effects of interacting factors on acoustic intervals of various sizes, e.g., effects of phrasal position on word-final acoustic intervals, where the largest effects occur on an acoustic interval corresponding to the phrase-final syllable rhyme (phrase-final lengthening), and acoustic intervals corresponding to word-initial onset closures (phrase-initial lengthening); effects of prominence (word-level and phrase-level stress) on syllable-sized intervals, and compression effects of the number of syllables in units such as words (particularly when the word is in phrasally prominent position), complex effects of overall speaking rate; and the interaction of all of these effects (and more) with segment-intrinsic durational patterns. Factors that affect intervals between acoustic landmarks can also affect characteristics of individual and coordinated movements, but do so in different ways, e.g., durations of movements toward consonantal constrictions are affected less by prosodic position than are more “steady state” regions. See Turk and Shattuck-Hufnagel (2020) for more detail about these effects and references.

Existing models of speech production vary in how many of these effects they can account for. Articulatory Phonology in the Task Dynamics framework (AP/TD) is the model which to date provides the most comprehensive coverage, and is one of the very few models which has accounts of multiple types of effects of prosodic structure on durational patterns. However, its phonology-intrinsic approach to timing is fundamentally different from that of other models, in large part because of its use of spatio-temporal phonological representations and its lack of a phonetic planning component that is separate from the phonology. In the AP/TD approach, such a component is not required because surface timing and spatial characteristics are emergent from the phonological component. This modeling approach contrasts with other models which have symbolic phonological representations, used to express categories of phonological contrast and phonological equivalence but do not specify spatio-temporal characteristics. As a result, these models have a phonetic planning component that is separate from the phonological planning component, to provide quantitative temporal, spatial, and spectral interpretations of the phonological representations. These differences among models lead us to ask a basic question: what is the most appropriate way to model systematic timing patterns in speech production?

The goal of this paper is twofold (1) to discuss the key features of existing articulatory models of speech production that govern their approaches to timing, along with advantages and disadvantages of each, and (2) to evaluate these features in terms of several pieces of evidence from both the speech and non-speech motor control literature. This evidence, taken together, supports an alternative approach to modeling speech production that is based on symbolic phonological representations and general-purpose, phonology-extrinsic, timekeeping mechanisms, rather than on quantitative spatio-temporal phonological representations and phonology-specific timing mechanisms. Thus, the evidence suggests that models in that alternative framework should be developed, so they can be tested with the same rigor as models based on spatio-temporal phonological representations with phonology-intrinsic timing mechanisms.

This paper is organized as follows: First, it presents key characteristics and differences among articulatory models that deal with timing issues, along with advantages and disadvantages of each. Second, it presents evidence from a wide variety of studies that bears on the appropriateness of these key characteristics, and the implications of this evidence for timing models. Third, it discusses Articulatory Phonology in the Task Dynamics framework, which to date is the most comprehensive, best-worked out model of timing, and why it is challenged by these findings. Finally, it discusses why the evidence supports 3-component models based on symbolic phonological representations and phonology-extrinsic timing, with separate components for phonological and phonetic planning, and motor-sensory implementation.

## Key Characteristics and Differences Among Articulatory Models That Deal With Timing Issues, Along With Advantages and Disadvantages of Each

### Spatio-Temporal vs. Symbolic Phonological Representations

Probably the most fundamental difference among current models of speech production planning has to do with the nature of phonological representations, which are symbolic in some, and spatio-temporal in others. Models with symbolic representations include Keating (1990), Fujimura (1992), Guenther (1995), and Henke (1966) et seq.; models with spatio-temporal representations include Articulatory Phonology (Browman and Goldstein, 1985, 1989, 1992; Saltzman et al., 2008; Goldstein et al., 2009) and its developments (e.g., Tilsen, 2013, 2016, 2018; Sorensen and Gafos, 2016; as well as Šimko and Cummins, 2010, 2011). It is important to note that although spatio-temporal representations in Articulatory Phonology are not symbolic, they are nevertheless abstract, because there is not a one-to-one mapping between phonological representations of each gesture and surface realization^1^.

The choice of the nature of phonological representations has fundamental implications both for the architecture of the speech production system and for the way it deals with timing issues. The dynamic spatio-temporal phonological representations of Articulatory Phonology “underlie[s] and give[s] rise to an action’s observable kinematic patterns” (Saltzman, 1995, p. 150). Therefore, although they are abstract, they include quantitative details that govern how speech articulations are produced in space and time in a given context (once gestural activation and overlap are specified in a gestural score). Thus, they make it possible to do without a separate phonetic planning component to provide these quantitative specifications. This appears advantageous, because it makes it possible for speakers (and listeners) to avoid “translating” from data structures in one component to data structures in another (Fowler et al., 1980). In addition, it makes it possible to avoid planning all of the quantitative details of speech production for each utterance: If the quantitative details (including timing) are represented in the phonological units and structures, speakers don’t need to explicitly plan them afresh for each utterance, in a separate phonetic planning component. Models with spatio-temporal phonological representations therefore have a very different architecture than those with symbolic phonological representations. That is, models with spatio-temporal representations typically have two components: (1) A single integrated component for both phonology and phonetics, and (2) a motor-sensory implementation component, whereas models with symbolic phonological representations typically have three: (1) A phonological planning component, (2) a separate phonetic planning component, and (3) a motor-sensory implementation component; in such 3-component models, the quantitative details of production are planned in the phonetic planning component.

Although obviating the need for complex online planning is a substantial advantage of the spatio-temporal approach, it is a challenge for this approach to provide an account of systematic contextual variability (including systematic timing variability) that is due to a range of factors such as overall rate of speech, prosodic position, segmental context, movement distance, etc. Existing spatio-temporal-based approaches have proposed additional mechanisms, such as adjustments to gestural activation time (Byrd and Saltzman, 2003; Tilsen, 2016), and/or additional, competing, target representations (Gafos, 2006; Gafos and Beňuš, 2006; see also Flemming, 2001^2^) to account for this variability. However, these approaches face the challenge of explaining how quantitative, spatio-temporal phonological representations and adjustments are learned, given that they are not directly observable from surface acoustics. In contrast, phonological learning is different in approaches with symbolic representations, where the learner must learn the phonological equivalence of variants that are members of a single category, but doesn’t have to infer quantitative parameter values that define the category from potentially ambiguous input^3^.

#### Emergent Surface Timing Characteristics vs. Explicitly Specified Surface Timing Characteristics

One of the critical implications of choosing spatio-temporal representations over symbolic representations is that models with spatio-temporal representations + adjustments of their activation can yield surface temporal patterns without having to explicitly specify surface timing characteristics in units such as milliseconds. This is because surface timing in these models is *emergent*, rather than explicitly specified. For example, in models that use mass-spring systems to accomplish movements toward constrictions, different surface duration patterns can be achieved by changing the stiffness of mass-spring systems without explicitly specifying a surface duration. Emergent systems would be advantageous if it turned out that surface durations are not represented; however, as will be argued below, there is evidence that surface durations are in fact represented. Furthermore, not representing surface durations of speech may make it difficult to interact with external events in the world, e.g., to finish an utterance before the occurrence of an anticipated event, expected to occur at a particular time.

#### Separate vs. Integral Specification of Spatial and Temporal Characteristics

Another characteristic that is implied by the choice of spatio-temporal phonological representations is that in these models, temporal and spatial characteristics are represented integrally in phonological representations. In contrast, in models with symbolic representations, which require a separate phonetic planning component, it is in principle possible to specify temporal characteristics separately from spatial characteristics. Integrated spatio-temporal representations would be advantageous if temporal patterns were predictive of spatial patterns, but would be challenged if, as is argued below, speakers are able to accomplish the same temporal pattern using different spatial paths of movement, particularly when a single speaker produces the same temporal pattern in more than one way.

### Use of General-Purpose, Phonology-Extrinsic Timekeepers and Timing Units vs. Phonology-Intrinsic Timekeepers

One might wonder whether it is in principle possible for models with spatio-temporal phonological representations to avoid the use of any type of timekeeper or timing unit. However, systematic contextual timing variability of speech (due to e.g., position-in-phrase, position-in-word, phrasal prominence, and speaking rate) appears to require timing control that specifies temporal extent. Thus to date all speech production models make use of some type of timekeeper, either a general-purpose timekeeper (in ms.) or a phonology-specific timekeeper. For example, Nam et al. (2010) use a phonology-specific timekeeper (gestural planning oscillators) to specify the relative timing of gesture initiation, and Saltzman et al. (2008) use such oscillators to specify the durations of gestural activation. In contrast, models with symbolic phonological representations assume a general-purpose timekeeper that operates with solar-timing units (e.g., ms). These include proposals by Fujimura (1992 et seq), Guenther (1995, 2016), and Henke (1966). Šimko and Cummins (2010, 2011)’s Embodied Task Dynamics is an example of an approach with spatio-temporal phonological representations that nevertheless assumes a general-purpose timekeeper and solar timing units. This approach provides an optimization account of systematic patterns of variability found in speech^4^. In this model, optimal movement parameters (including the duration of gestural activation as measured in milliseconds) are determined on the basis of several movement costs (effort, parsing, and time), where the time cost is based on utterance duration as measured in solar time units.

It would be difficult to distinguish models with phonology-specific and general-purpose timekeepers if the timing units in both types of models were linearly related. However, mechanisms for lengthening gestural activation intervals that involve slowing the phonology-specific clock (e.g., Pi and Mu_*T*_ gesture adjustments, Byrd and Saltzman, 2003; Saltzman et al., 2008) warp the relationship between phonology-specific time and solar time in parts of utterances that are affected by Pi and Mu_*T*_ gestures, such as boundary-adjacent intervals and stressed syllables. That is, in models that use phonology-specific “clock”-slowing to accomplish boundary- and prominence-related lengthening, the lengthened intervals do not contain more phonology-specific units, although they are longer in solar time, warping the relationship between these two kinds of representations in non-linear ways across an utterance, and in inconsistent ways between utterances. Diagnostics of speakers’ representations of the durations of boundary-related and/or prominence-related intervals would provide a way of determining whether phonology-specific vs. solar timing units are more appropriate; see section “Constraints on Lengthening Due to Phrasal Prosody Suggest That Surface Timing Patterns Are Represented, and Not Emergent” for evidence that bears on this issue.

### Different Ways of Modeling the Time Course of Individual Movements

Models of speech production also differ in the mechanisms they use for achieving constriction-related movements that have appropriate movement velocity profiles. In Fujimura’s (1994) model, movements toward constrictions, called “elemental gestures,” are modeled as impulse response functions, parameterized for various aspects of the movement timecourse (i.e., affecting the shape of the velocity profile) as well as inherent amplitude. The values of the parameters for each elemental gesture are stored in a table. As long as the gestures are not constrained by e.g., saturation effects, the parameter values in the table are modified in a produced utterance according to a modification factor (the syllable pulse) that represents each syllable’s strength in an utterance. In this model, elemental gestures for vowels change slowly over time, and faster-changing consonantal gestures are superimposed on these.

In Articulatory Phonology in the Task Dynamics framework, gestural movements are generated using a second order mass-spring system with a linear restoring force. The point attractor mass-spring dynamics of this model appropriately generates a smooth, single-peaked tangential velocity profile, i.e., with a single acceleration and a single deceleration phase. However, the velocity profiles generated by systems with linear restoring forces are asymmetrical, with velocity peaks that are earlier than observed in empirical data. To create more realistic velocity profiles, gestural activation functions which originally were turned on and off abruptly, were instead shaped to have gradual activation interval on- and off-ramps, and these were shown to successfully generate velocity profiles with centered peaks (Byrd and Saltzman, 1998). More recently, Birkholz et al. (2011) and Sorensen and Gafos (2016) showed that other types of mass-spring systems could generate more realistic timing of the velocity peak without gradual on-and off- ramps for gestural activation. Birkholz et al. (2011) used a 10th order linear mass-spring system, and Sorensen and Gafos (2016) used a second order system with a non-linear restoring force. Sorensen and Gafos (2016) showed that their system with a non-linear restoring force had the added advantage of providing an account of the observation that longer distance movements are longer in duration than shorter distance movements, in spite of higher peak velocity (cf. Fitts, 1954 law).

Movement trajectories (and consequently their velocity profiles) are generated in a different way in Guenther’s DIVA model (2016). This model generates articulatory movement trajectories via a neural network mapping between directions in sensory space and velocities of articulators (Guenther and Micci Barreca, 1997; Guenther, 2016). In this model, articulatory movement trajectories are generated which produce acoustics that fall within a spectro-temporal target template for each speech sound. Thus, the time course of movement is determined by the way acoustic formants vary over time, and not by any explicit motor principle.

The non-speech motor control literature has proposed other ways of modeling appropriate velocity profiles. Nelson (1983), Harris and Wolpert (1998), and Tanaka et al. (2006) present Optimal Control Theory accounts. For example, Tanaka et al. (2006) propose that movements are produced with minimum durations that conform to accuracy requirements, and show that appropriate velocity profiles and movement durations can be generated for different accuracy requirements on the assumption that noise grows with the size of the neural control signal. Harris and Wolpert (1998) and Tanaka et al. (2006) successfully predict the relationships among speed, distance, and accuracy described in Fitts’ (1954) law.

Lee (1998) proposes that movement velocity profiles are governed by tau-coupling, where tau = time-to-goal-achievement at the current movement rate. Appropriate movement velocity profiles can be generated if actors keep their taus τ_**X**_ in constant proportion to the taus of a Tau Guide τ_**G**_, by making τ_**X**_ = *K*τ_**G**_, where $\tau_{G}=\frac{1}{2}\,\left(t-\frac{T^{2}}{t}\right)$, *t* is time and *T* is movement duration (The equation is based on Newton’s equations of motion). The value of the coupling constant *K* determines the skewness of the velocity profile. If *K* = 1, the movement accelerates at a constant rate; lower values of *K* have an acceleration followed by a deceleration, with longer decelerations for lower values of *K*. Lee’s model has the advantage of being computationally simpler than Optimal Control Theory accounts. It predicts that actors should be able to manipulate velocity profile skewness via the *K* parameter. This provides a potential account of velocity profile skewness differences observed in the non-speech and speech motor control literature (e.g., Perkell et al., 2002). For example, a bird attempting to land on a twig will have an earlier velocity peak to ensure a gentle, accurate, low velocity landing, whereas a tongue approaching the roof of the mouth for a /t/ might have a later velocity peak.

### Different Ways of Modeling Coordination

Another way in which articulatory models of speech production differ is in the ways that they model the temporal coordination of articulatory movements. Coordination can be described at different levels, including the coordination of movements that contribute to a single constriction, as well as the coordination of movements that contribute to sequences of constrictions. Models differ on the information used to determine relative timing patterns, i.e., on whether they are based on relative timing vs. spatial characteristics vs. absolute timing. For example, in Fujimura’s model, where faster consonantal gestures are superimposed on slower, vocalic gestures, coordination is based on relative timing: Consonantal elemental gestures are triggered at appropriate delays or lags from the syllable pulse, where the delays are specified as ratios of the syllable duration (Fujimura, 1994; Wilhelms-Tricarico, 2015).

Nam et al.’s (2010) theory of coupled oscillator model of coordination is also based on relative timing, that is, one constriction formation gesture is initiated when a particular planning oscillator phase of an earlier gesture is reached. On this view, if coupled planning oscillators speed up or slow down, the relative timing of gestures governed by the oscillators will be preserved. While Tilsen (2016) adopts this relative timing view for the coordination of onset consonants with syllable nuclei, he proposes a different mechanism based on spatial characteristics for the coordination of coda consonants with syllable nuclei. Tilsen (2016) proposes that coda consonant gestures are activated at the achievement of nucleus gestural target, i.e., on the basis of spatial information. In contrast, Šimko and Cummins’ model proposes that gestural coordination and overlap are governed by costs of parsing (perceptual recoverability) and absolute time. For example, among other things, a higher parsing cost will encourage the speaker to make gestures more perceptually recoverable by making them less overlapped, and a higher time cost will make utterance duration shorter through increased overlap.

Whereas models of speech production have to date focused primarily on the relative timing of movement initiation, models available in the non-speech motor control literature suggest another possibility, namely coordination based primarily on the goal-related parts of movement, where movements are initiated at a time that ensures spatial and/or temporal accuracy (Harris and Wolpert, 1998; Todorov and Jordan, 2002; Tanaka et al., 2006). Similarly, on Lee’s (1998, 2009) view, movements are controlled based on tau-coupling (tau = time-to-goal-achievement at the current movement rate) to achieve their goal at a particular time. Thus, his model ensures synchronous goal achievement for all movements that are tau-coupled before the end of movement, but does not require that these movements begin synchronously. See Turk and Shattuck-Hufnagel (2020) for more discussion of Lee’s General Tau Theory as applied to speech.

### Different Ways of Modeling Effects of Prosodic Structure on Timing

In spite of growing evidence that prosodic structure has a systematic influence on the durational patterns of virtually all known languages, relatively few articulatory models have explicit accounts of these and other contextual effects on timing. Here, we discuss models which have explicitly modeled prosodic effects in different ways.

Fujimura (1992 et seq.) framework assumes that phonological representations are expressed in terms of symbolic distinctive features, as well as symbolic representations of syllables (including their sub-constituents, i.e., onsets, nuclei and codas), and assumes higher-level prosodic constituency which can influence syllable durations in the vicinity of higher-level constituent boundaries. The syllable representations are mapped onto a “syllable pulse train,” i.e., a series of (usually symmetric) triangles corresponding to syllables and pauses (if they occur), whose bases are contiguous. Triangle heights represent an appropriate magnitude multiplication factor (the pulse) which controls syllable prominence and phrasal boundary effects, and triangle bases represent syllable or pause duration. As a default, the apex angle is assumed to be the same for all triangles; therefore syllable triangle height correlates with syllable duration, so that longer duration and prominence are linked. In cases where additional lengthening is required, either the apex angle can be adjusted, or additional (half) triangles can be added to the utterance (Fujimura, 2002). Although this model provides a framework for modeling the influence of prosodic structure on correlated spatial and durational characteristics, it doesn’t provide a way of determining what the syllable pulse heights and apex angles (and hence the syllable durations) should be for a given context.

Šimko (2009), Šimko and Cummins’s (2010, 2011), and Windmann’s (2016) approaches are of note in this regard, because they propose a principled cost-minimization mechanism for determining durational properties of speech, based on Optimal Control Theory. Šimko and Cummins’ Embodied Task Dynamics model is a development of the Task Dynamics model used in AP/TD, in which the articulators are assigned masses, and optimization is used to determine model parameter values. In this model, gestural activation onset and offset timing (specified in solar time units) and system stiffness (where system stiffness is a scaling factor for gestural and “speech-ready” stiffness^5^) are optimized using three costs: An effort cost, a perceptual (parsing) cost, and a time cost which is a linear function of utterance duration in milliseconds. Beňuš and Šimko (2014) showed that locally decreasing the duration cost in the vicinity of a phrase boundary can be used to model boundary-related lengthening in Slovak *m(#)abi* and *m(#)iba* sequences^6^.

Although Šimko and Cummins’ approach is based on Articulatory Phonology, it differs from AP in the use of solar timing units, which are used for the specification of its time cost, as well as for the specification of gestural activation durations which result from their optimization procedure. In contrast, Articulatory Phonology in the Task Dynamics framework (Byrd and Saltzman, 2003; Saltzman et al., 2008) provides an approach in which solar timing units are not required, and where surface timing patterns are fully emergent from phonology-specific processes. In their approach, lengthening effects due to prosodic structure are modeled as adjustments of gestural activation durations. Gestural activation durations are not specified in milliseconds, but rather in proportions of gestural planning oscillator periods. At a default rate of speech, gestural activation duration corresponds to gestural mass-spring settling time, i.e., the time required for a gesture to approximate its target. In particular prosodic positions, such as phrase-boundary-adjacent position, or at slower speech rates, the default gestural activations are stretched (Byrd and Saltzman, 2003). This stretching is implemented in later versions of the theory (Saltzman et al., 2008) by slowing the frequency of the gestural planning oscillators. Analogously, at faster rates of speech, or in unstressed positions, the default gestural activations are shortened by speeding up the frequency of the gestural planning oscillators. This approach has been used successfully to model effects of prominence, boundary-adjacency, and poly-sub-constituent shortening.

Tilsen’s recent development of AP (Tilsen, 2018) provides another mechanism for prominence-related lengthening, based on feedback about target approximation. In this model, one mechanism for ending gestural activation is the suppression of gestural activation after targets are approximated. In this proposal, prominent syllables and syllables produced at a slow speech rate are proposed to result from a high degree of reliance on external feedback about target approximation.

## Evidence From the Literature That Relates to These Characteristics and Constrains the Choice of Appropriate Model

The previous section showed substantial differences among existing models of speech articulation control timing patterns. Many of these differences derive from choices about the general architecture of the system and about the nature of phonological representations that encode contrast, phonological equivalence and prosodic structure. In spite of the differences, these models all generate plausible articulatory trajectories, at least in some contexts. How can they be distinguished? In this section, we discuss phenomena which bear on this question, focusing on the issues of (1) emergent vs. specified surface timing patterns (2) spatio-temporal representations vs. the independent representation of timing information (3) the use of phonology-specific vs. general-purpose timekeepers, (4) spatio-temporal representations vs. symbolic representations, (5) movement coordination, and (6) modeling effects of prosodic structure.

Evidence bearing on these issues motivates an alternative approach to modeling timing control, i.e., a phonology-extrinsic approach based on symbolic phonological representations in a Phonological Planning Component, with specifications for surface durations that are planned in a Phonetic Planning Component that is separate from the Phonological Planning Component. The first two phenomena, (1) constraints on lengthening due to phrasal prosody, and (2) different strategies for controlling rate of speech, boundary-related lengthening and quantity, suggest that surface durations are explicitly represented. As a result, they present a challenge to approaches to timing in which surface durations emerge without explicit representation; moreover, the second phenomenon suggests that surface durations can be specified independently of spatial characteristics, since the timing patterns are the same while the spatial characteristics vary. The third phenomenon, (3) more timing variability for longer duration intervals in speech and non-speech behavior, suggests the involvement of a noisy general-purpose timekeeping mechanism in the speech production process, in which longer durations intervals are associated with more timing variability due to accumulated noise. Finally, the fourth phenomenon (4) less timing variability at movement endpoints compared to other parts of movement, challenges the concept of spatio-temporal representations, and suggests that movement coordination is based on goal-related parts of movement rather than onsets. Taken together, these four phenomena support the alternative view that speech production planning is based on symbolic phonological representations and includes separate components for Phonological and Phonetic Planning, as well as a third, Motor-Sensory Implementation component in which speech movements and acoustics are monitored and adjusted to ensure that spatial and timing goals are achieved appropriately (Houde and Nagarajan, 2011; Guenther, 2016).

### Constraints on Lengthening Due to Phrasal Prosody Suggest That Surface Timing Patterns Are Represented, and Not Emergent

**FIGURE 1:**
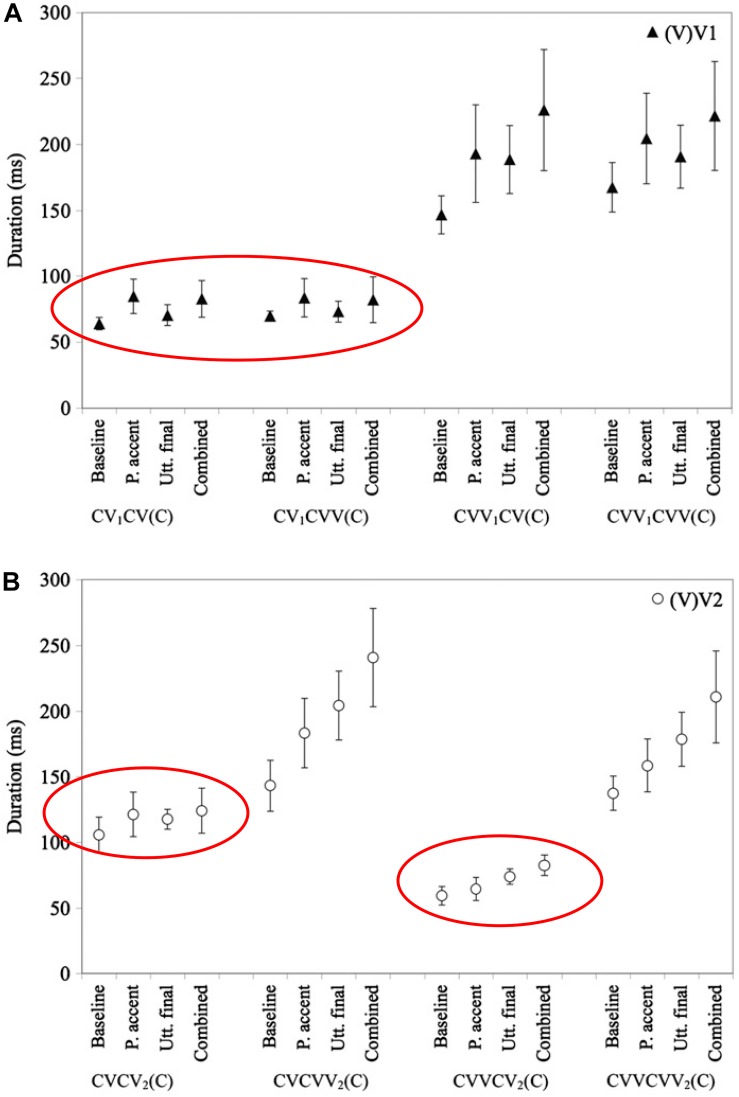
Based on a similar figure in Nakai et al. (2012), with ellipses added. Reprinted by author and publisher permission. Caption as in the original: “Mean test vowel durations (in ms) in the baseline and three experimental conditions (P. accent = phrasal accent, Utt. final = utterance final, Combined = Combined-effect). The durations of (V)V1 [i.e., vowel in the first vowel] are plotted in the upper panel **(A)**; (V)V2 [i.e., vowel in the second syllable] in the lower panel **(B)**. Error bars represent ± 1SD.” Values for phonemically short vowels are circled in both panels.

In Northern Finnish and Dinka, which have a phonemic quantity contrast, the phonemically short vowels are lengthened less than the long vowels, in prosodic contexts such as phrase-final position (Remijsen and Gilley, 2008; Nakai et al., 2009, 2012). For example, as Figure 1 shows, the magnitude of final, accentual, and combined lengthening of phonemically short vowels in Northern Finnish is restricted compared to lengthening on phonemically long vowels (Nakai et al., 2009, 2012). This suggests that speakers of this language explicitly constrain the surface durations of phonemically short vowels to maintain the duration contrast with longer vowels.

In this figure, VV refers to a phonemically long vowel, and V to a phonemically short vowel. Note that in the last syllable of CVCV(C) words, cf. the left-hand side of Figure 1B, the phonemically short vowel shows a greatly reduced magnitude of combined accentual + final lengthening (17%) compared to the phonemically long vowel in the same context (68%). The lengthening pattern on this so-called “half-long vowel”^7^ is suggestive of a constraint resulting in a surface duration of phonemically short vowels of < ca. 140 ms, at least at this speaking rate, supporting the view that the (phonemically short) half-long vowels are lengthened less than the long vowels to avoid endangering the phonemic contrast between short and long vowels in this language. Two types of empirical evidence for a constraint on the surface duration of phonemically short vowels are provided in Nakai et al. (2009, 2012). First, Nakai et al. (2009) found a negative correlation between phrase-medial duration and the amount of final lengthening for V2 in CV1CV2 structures. One might initially imagine a mechanism by which speakers could learn to lengthen phonemically short vowels less to avoid confusion in their listeners, without explicitly representing a durational constraint. However, this potential solution is ruled out by the observation that speakers adjust the amount of lengthening for their phonemically short vowels in a way that maintains a surface durational distinction. That is, phonemically short vowels that are shorter are lengthened more, and phonemically short vowels that are longer are lengthened less, showing evidence of a surface duration constraint. Further support for a surface duration constraint comes from Nakai et al. (2012)’s study of final lengthening and accentual lengthening, which combine sub-additively for V2 in CV1CV2. A constraint on lengthening of this type is difficult to express in a system that does not explicitly represent surface durations.

**FIGURE 2:**
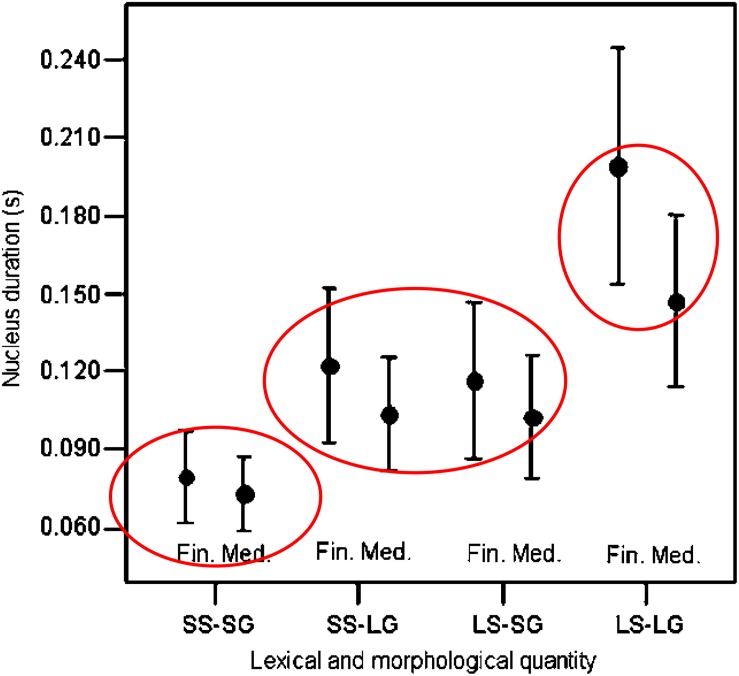
Based on a similar figure in Remijsen and Gilley (2008), with ellipses added. Reprinted by author and publisher permission. Original caption: “Means and standard deviations for vowel duration as a function of lexical/morphological quantity—short stem in short grade (SS-SG), short stem in long grade (SS-LG), long stem in short grade (LS-SG), long stem in long grade (LS-LG)—and sentence context—(Medial, Final). Items ending in /r/ are excluded.” SS-SG are considered to have short quantity, SS-LG and LS-SG are considered to have medium quantity, and LS-LG are considered to have long quantity. Values for short, medium, and long quantities are separately circled.

The final lengthening patterns in Dinka, a Nilotic language, are also consistent with this type of constraint. This language has a three-level quantity system, and vowels of short and medium quantities are lengthened less than the long vowels, in phrase-final position, a prosodic context that requires duration lengthening (see Figure 2, reproduced from Remijsen and Gilley, 2008).

The results reviewed here suggest that the explanation relates to surface durational information which is represented in the minds of speakers, and is involved in the maintenance of phonemic contrasts. These results are difficult to account for in models in which surface durations are the emergent output of activation interval durations + phonology-intrinsic clock-slowing adjustments, are not explicitly represented, and so cannot be invoked as constraints on lengthening.

### Different Strategies for Manipulating Durations (in e.g., Rate of Speech, Boundary-Related Lengthening, and Quantity), Suggest That Surface Timing Goals Are Explicitly Represented, and Not Emergent

The explicit representation of surface duration requirements is supported by another type of evidence, related to the implementation of overall rate of speech as well as to boundary-related lengthening, and to duration-related quantity differences. This evidence suggests that speakers specify surface interval duration requirements as goals of speech production, and meet these requirements using a variety of different strategies. The equivalence of these strategies goes unexplained in theories that cannot represent surface durations. That is, the only thing shared by all of the different strategies is their equivalent effects on surface durations.

One example of this kind of evidence is that, when asked to speak quickly, speakers achieve surface durations using a wide variety of strategies. Although it is often the case that speakers accomplish this task by reducing the number and/or durations of pauses at fast rates, other strategies have also been observed. For example, acoustic studies show that speakers may manipulate the number of pauses, but not the durations, or vice versa (Fletcher, 1987; Trouvain, 1999). Likewise, kinematic studies reveal that, although the peak velocity/distance relationship for movements is often higher at fast rates, some speakers achieve faster rates by increasing articulatory speed (peak velocity), while other speakers achieve this by reducing movement distance (Abbs, 1973; Ostry and Munhall, 1985; Engstrand, 1988; Goozée et al., 2003; see Berry, 2011 for a review). And while many speakers show increased articulatory overlap at fast rates, not all speakers do (Engstrand, 1988; Boyce et al., 1990; Shaiman et al., 1995; Byrd and Tan, 1996; Shaiman, 2001, 2002; all cited in Berry, 2011).

What these studies show is that speakers respond differently to instructions when asked to speak quickly, but in all cases achieve shorter utterance durations. We cannot see how the equivalence of their strategies can be expressed without reference to the surface duration goals of these utterances. Similar findings of different strategies for achieving similar surface duration goals have been observed for quantity differences and phrase-final lengthening (Edwards et al., 1991; Hertrich and Ackermann, 1997). Hertrich and Ackermann’s (1997) findings are of particular note because they show that the same speaker can use different strategies to achieve durational differences in different contexts. For example, Hertrich and Ackermann (1997) showed that the same speaker used different strategies to achieve the phonemically short vs. long distinction for different vowels. That is, some speakers showed a longer opening movement for /ɑ:/ compared to /ɑ/, but a predominate pattern of a longer initial part of the closing movement for /u:/ compared to /u/. Similarly, Edwards et al. (1991) showed that the same speakers used different strategies for achieving longer surface durations in phrase-final position (compared to phrase-medial position) at different rates of speech. That is, at faster rates, they slowed articulatory speed in phrase-final position, but at a slow rate, they kept speed constant and held the articulators in quasi-steady states.

Taken together, these studies of strategies for adjusting durations for rate of speech, vowel quantity, and final lengthening suggest that surface durations are speech production goals that can be achieved in a variety of ways. This type of motor equivalence supports the view that (1) surface duration requirements can be specified as part of the speech production process, and (2) these durational requirements or goals are separately specified from how the goals are achieved. Particularly telling are cases where the same speakers show different articulatory strategies for achieving similar durational patterns in different speaking-rate contexts.

These findings support models in which (1) surface duration goals (or costs) for intervals can be explicitly represented during phonetic planning, and (2) these goals are specified separately from how the goals are achieved articulatorily. This type of model architecture would make it possible for the same goal to be achieved in a variety of ways.

### More Timing Variability for Longer Duration Intervals Suggests the Involvement of General-Purpose Rather Than Phonology-Specific Timekeeping Mechanisms

The previous sections presented evidence suggesting that speakers explicitly represent surface timing goals, and can accomplish those timing goals in many different ways. We argued that emergent timing mechanisms specific to the task of speaking cannot account for the observed behavior, raising the question of what kind of alternative mechanism could support the planning of such intervals. This section presents evidence from timing variability that suggests an answer: general purpose timing mechanisms that could be used in specifying and planning surface durations in speech.

Many types of timed behaviors show what is known as “the scalar property,” a relationship between interval duration and variability that tends to follow Weber’s law, resulting in an approximately constant coefficient of variation (SD/mean) over a range of intervals. Getty (1975) proposed that variability in interval durations arises from two sources of noise (1) a duration-dependent source, thought to be the consequence of noise in a timekeeping process, and thus to increase with the duration of the interval, and (2) a source of variability due to noise in the motor system, assumed to be constant regardless of the duration of the interval. This proposal provided an account of the higher coefficient of variation (SD/mean) observed for shorter intervals (up to approximately 200 ms) as compared to longer intervals (approximately 200–1300 ms). For a review of different modeling approaches to general purpose timekeepers with accounts of timing variability, see Schöner (2002).

Behaviors showing timing variability that grows with interval duration include:

• Periodic non-speech tasks such as tapping and periodic elbow flexion, back-and-forth line drawing, and periodic circle drawing. These tasks typically involve moving to a metronome and then continuing the periodic activity without the metronome. Measurements used in assessing the relationship of timing variability to interval duration are typically made from the continuation phase.
• Non-periodic non-speech behavior tied to the anticipated arrival of a stimulus (for human and non-human animals, Gibbon, 1977; Roberts, 1981; Green et al., 1999; and many others), as well as the production of single timed intervals (Rosenbaum and Patashnik, 1980; Ivry and Hazeltine, 1995; Merchant et al., 2008).
• Non-periodic speech tasks, where longer duration movements and intervals at phrase boundaries show more variability than phrase-medial intervals. For example, Edwards et al. (1991) and Byrd and Saltzman (1998) show greater variability for longer duration movements at phrase vs. word boundaries, and Gafos et al. (2014) show more variability for longer duration intervals between consonantal events in consonantal clusters (e.g., first consonant target-to-second consonant release). Similar findings are reported in the speech literature for intervals measured from landmarks (Stevens, 2002) in the acoustic signal. For example, phonemic quantity differences (Dinka: Remijsen and Gilley, 2008; N. Finnish: Nakai et al., 2012); Chen (2006) for focused vs. non-focused constituents in Mandarin; Nakai et al. (2012) for final and phrasally accented vs. non-final, non-accented intervals in N. Finnish; and Lefkowitz (2017) for a linear relationship between standard deviation and mean duration of vowel intervals across a very wide range of contexts in an English experiment.

Findings of greater timing variability in phrase-final and/or phrasally prominent positions are thus consistent with the view that speech makes use of a general-purpose timekeeping mechanism, with variability that is proportional to the surface duration of the timed interval, as suggested by Gallistel, 1999; Gallistel and Gibbon, 2000; Jones and Wearden, 2004; Shouval et al., 2014; and others). The law applies to timing behavior in many different tasks (non-speech) and speech, and in perception and in production. Whatever mechanism accounts for this law therefore appears to be general across all of these tasks and behaviors. General purpose timekeeping mechanisms thus provide a unified account of timing variability for all timed intervals; see below for further discussion.

### The Observation of Less Timing Variability at Movement Endpoints Than at Other Parts of Movement Challenges (Spatio-)Temporal Phonological Representations, and Supports a Model of Speech Production Based on Symbolic Phonological Representations

The sections above presented three types of evidence for the representation of surface time intervals in the planning of movements – a constraint on the surface durations of phonemically short vowels in some quantity languages; multiple articulatory strategies for attaining appropriate acoustic durational patterns, suggesting that those patterns themselves are the goals of the movement, and the increase in variability with longer intervals, suggesting that those intervals are generated using a general purpose phonology-extrinsic timing mechanism that operates in units of surface (solar) time, rather than in phonology-intrinsic timing mechanisms operating in non-solar time units. In this section we present an argument for symbolic (as opposed to spatio-temporal) phonological representations. The evidence for this argument comes from observations of less timing variability at particular parts of movement, which are most behaviorally meaningful. This evidence supports symbolic representations because it requires a representation of the most behaviorally meaningful part of movement so it can be prioritized for timing accuracy. It supports symbolic representations because they can map onto a part of movement that relates most directly to the achievement of a phonological goal, and can therefore be prioritized. Such symbolic representations require the specification of timing and other phonetic characteristics in a separate phonetic planning component. Thus, this evidence for symbolic phonological representations provides a fourth argument for the use of phonology-extrinsic time, because there are no time specifications in the phonology.

Evidence for the representation of individual parts of movement, so that their timing can be prioritized, comes from a number of sources. In his 1998 paper, Dave Lee notes: “it is frequently not critical when a movement starts – just so long as it does not start too late. For example, an experienced driver who knows the car and road conditions can start braking safely for an obstacle a bit later than an inexperienced driver.” This observation suggests that the timing of the part of movement most closely related to the goal attainment should be less variable than the timing of other parts of movement^8^. This section presents evidence from repeated movements elicited in controlled laboratory experiments that that confirms Lee’s prediction.

Many findings in the literature are consistent with the observation that the timing of movement endpoints can be less variable than for other parts of a movement, even for repeated movements that have the same movement path^9^. For example, Gentner et al. (1980) study of keypress timing in typing found lower consistency in the start times of key press movements, as compared to the end times, for two typed repetitions of the same sentence, performed by an experienced typist. The median difference in start times was 58 ms, compared to a difference of 10 ms for end times.

Additional evidence for lower timing variability at movement endpoint can be found in periodic tapping data (Billon et al., 1996; Spencer et al., 2003; Zelaznik and Rosenbaum, 2010), For example, Spencer et al. (2003) found that timing variability in repetitive tapping showed lower variance at finger touchdown as compared with the time of peak velocity. Zelaznik and Rosenbaum (2010) found similar results for tapping, in that timing variability of contact with the tapping surface was lower than that of maximum finger extension. Interestingly, however, both Spencer et al. (2003) and Zelaznik and Rosenbaum (2010) found a different pattern of results for circle drawing, that is, no evidence for differences in timing accuracy at different points in the circle cycle. For example, in Zelaznik and Rosenbaum (2010), the variability at cycle onset (0°) was no different from timing variability at a spatial location opposite to cycle onset (180°). This evidence is consistent with the emergent timing view of continuous circle drawing, that is, that timing in such tasks is primarily emergent from dynamic characteristics and has minimum involvement from a timekeeping mechanism. See Zelaznik and Rosenbaum (2010) and Studenka et al. (2013) for evidence less consistent with emergent timing for circle drawing when it creates a perceptual (auditory or tactile) event that could be thought of as the goal of the movement, consistent with the idea that when salient timing-related events can be identified, general-purpose timing mechanisms are likely to be invoked; see Repp (2008) and Repp and Steinman (2010) for more nuanced discussions.

**FIGURE 3:**
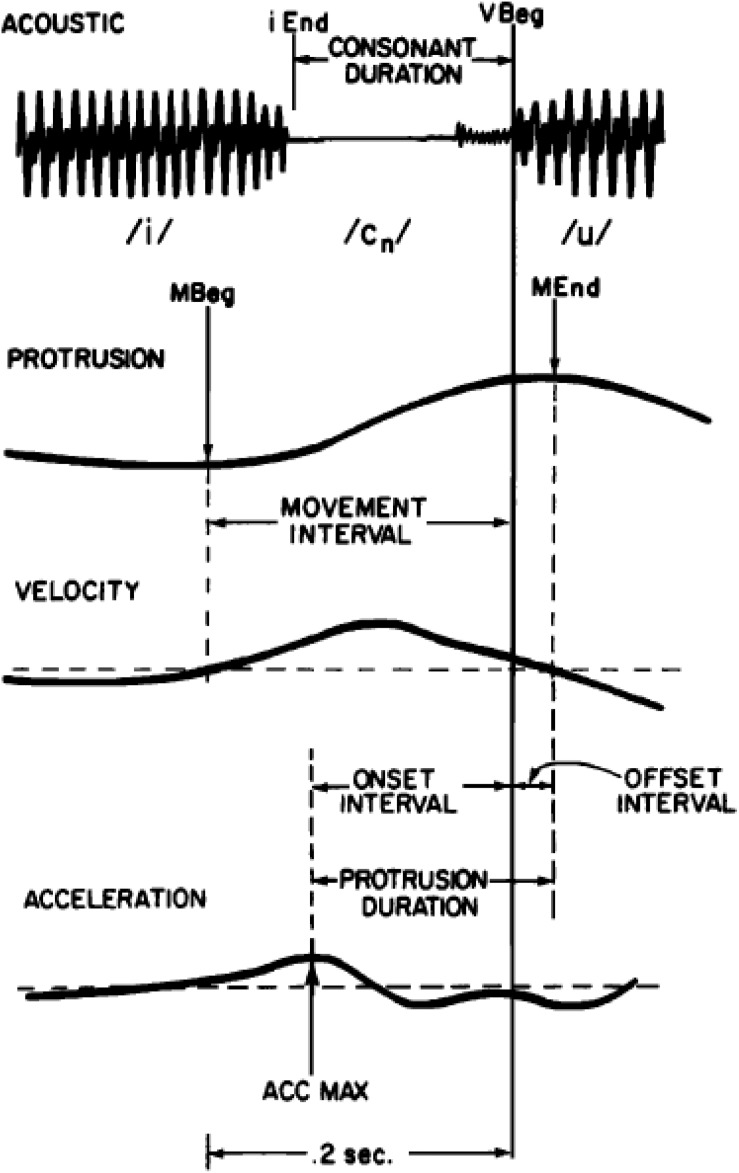
Reproduced from Perkell and Matthies (1992), with the permission of AIP Publishing. Caption as in the original: Schematic illustration of data extraction. From top to bottom: (1) a segment of the acoustic signal (ACOUSTIC), (2) lip protrusion (PROTRUSION), (3) lip velocity (VELOCITY), and (4) lip acceleration (ACCELERATION) vs. time. Acoustic events in the time-expanded acoustic signal are end of the /i/ (iEnd) and beginning of the /u/ (Vbeg). Movement events are: movement beginning (mBeg), movement end (mEnd), and maximum acceleration (AccMax).

**FIGURE 4:**
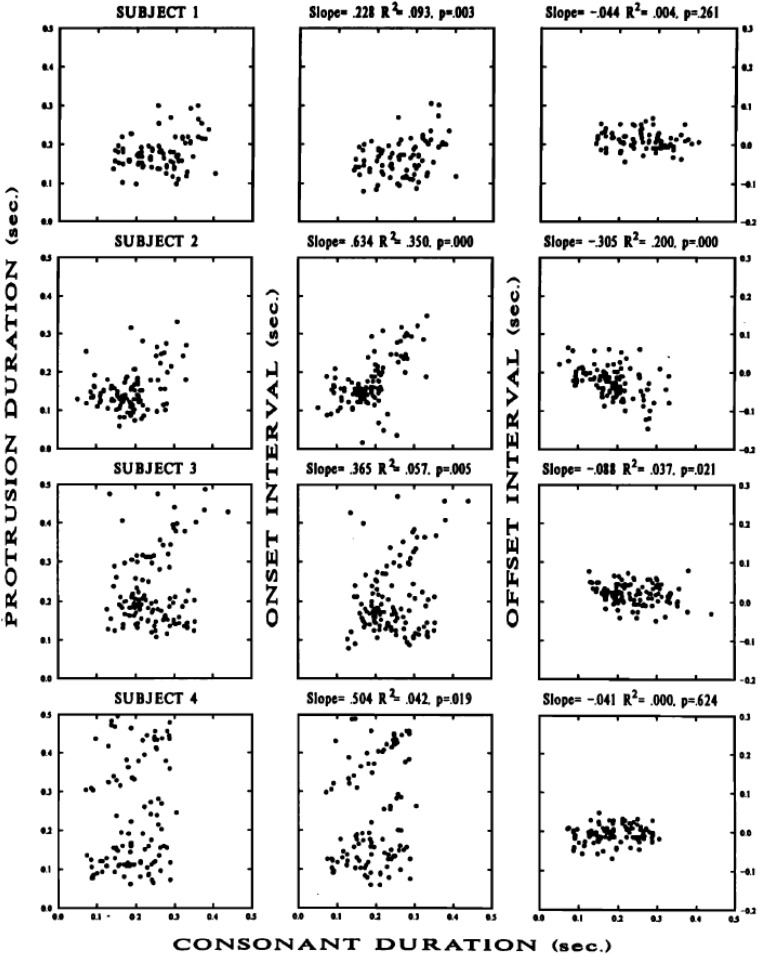
Reproduced from Perkell and Matthies (1992), with the permission of AIP Publishing. Scatter plots of protrusion duration interval vs. consonant duration (**left** column); onset interval vs. consonant duration (**middle** column), and offset interval vs. consonant duration (**right** column) for lip protrusion movements from four participants’ /i_u/ sequences (shown in each of four rows). Details in Perkell and Matthies (1992).

Although speech production data on this topic is limited, the available data show timing variability patterns that are consistent with those observed for typing and periodic tapping; that is, they show less timing variability at goal-related parts of movement, such as movement endpoint, than at other parts of movement, such as the movement onset. Because it is often difficult to accurately diagnose movement onset times for a particular articulator when its movements may have been governed by multiple phonemes, Perkell and Matthies (1992) studied timing variability for upper lip protrusion movements during spoken /i_u/ sequences, where intervening consonants were /s,t,k/ and /h/, which are not normally associated with upper lip movement. The number of intervocalic consonants was varied systematically. Furthermore, to be sure that these intervening consonants did not have upper lip movement associated with them, they carefully examined upper lip protrusion traces during /i_i/ contexts. Because they observed that /s/ did in fact have some idiosyncratic upper lip movement associated with it during the production of /isi/, they removed data with intervening /s/ from their analysis. As an additional precaution to ensure that their measure for movement onset was under sole control of the following /u/, they identified movement onset not as a point of velocity zero, but as the point of acceleration maximum, i.e., a time point clearly associated with movement toward the /u/ target. After all of these precautions to ensure that the measured upper lip protrusion timing data was due to the production of /u/ alone, they still found more variability for the timing of acceleration maximum as compared to the timing of maximum protrusion. As shown in Figures 3, 4^10^, they observed lower variability in the timing of movement endpoint (maximum protrusion) relative to voicing onset for /u/, as compared to the timing of maximum acceleration, relative to voicing onset for the same vowel. This pattern suggests a tighter temporal coordination of maximum lip protrusion (movement endpoint) with voicing onset than of lip protrusion movement onset (max. acceleration) to voicing onset, and suggests that the timing of movement endpoint has higher priority than the timing of movement onset in these speech movements. This pattern suggests that having maximally protruded lips at the onset of voicing is the prioritized goal.

The view that the timing of the most behaviorally meaningful part of movement is given highest priority is supported by Gafos et al. (2019) evidence relating to the coordination of consonant clusters [bd, db, dg, gd, br, rb, kr, rk, kl, lk, lb, and nk] in three different positions-in-word in Moroccan Arabic. This evidence shows that movements toward C2 exhibit higher amplitude-normalized peak velocity the later they begin relative to C2 release. This finding supports the idea that speakers ensure appropriate movement velocity in order to achieve the behaviorally meaningful part of C2 (possibly its release) on time.

Additional evidence for the prioritization of timing accuracy at goal-related parts of movement can be found in speech-related manual gesture (Leonard and Cummins, 2011). They found less timing variability at a point of maximum hand + arm extension compared to other parts of movement, for hand + arm “beat” gestures that co-occur with speech. They recorded hand + arm movements (by recording the movement of an LED marker attached to the base of the thumb) while a speaker read two repetitions of three short fables. They found that the point of maximum extension of the hand before retraction had the least timing variability compared to other parts of movement (movement onset, peak velocity of extension, peak velocity of retraction and movement retraction end), measured relative to landmarks in the stressed syllable in each word. These findings suggest that the point of maximum extension is the part of movement which is coordinated with stressed syllables, as opposed to the onset of movement.

Taken together, these results suggest that particular part(s) of movement can be more task-relevant, or “behaviorally meaningful” than other parts of movement (cf. Shaffer, 1982; Semjen, 1992; Billon et al., 1996, for timing). They are also consistent with the view that the most task-relevant features of motor performance are prioritized for accuracy and therefore have the least variability, as proposed in Todorov and Jordan’s (2002, 2003) Minimal Intervention principle (cf. Winter, 1984; Lacquaniti and Maioli, 1994; Scholz and Schöner, 1999; discussed in Scott, 2004). Semjen (1992) makes this point about the control of finger movements in typing: “When copying a text, the typist probably attempts to produce the successive keystrokes fluently and at a fast sustained rate. The typist would thus anticipate the temporal properties of a sequence of behaviorally meaningful events, rather than the characteristics of the individual movements producing them. … We are thus led to a notion of multi-level temporal organization in serial movements, with some level(s) being more directly related to the subject’s intentions than others.” (Semjen, 1992, p. 248).

Along these lines, findings of greater temporal accuracy at particular parts of movement suggest that these parts of movement are “behaviorally meaningful” and are more closely related to the speaker’s goals for the utterance. For example, the various movements of the articulators must be coordinated to create particular configurations at appropriate times, or the goal of signaling the features, sounds and words of the utterance will not be met. Other, less behaviorally meaningful parts of movement are produced in service of achieving those goals.

The finding that goal-related parts of movement are more accurate/less variable than other parts of a movement requires the representation of a movement goal as separate from the way the movement is achieved, as well as a mechanism to ensure more precise timing accuracy at the goal-related part of movement. As discussed below, this challenges models with spatio-temporal representations in which there is no distinction between the goal of a movement and the way it is achieved, because without this distinction, the phonological representation (and thus the phonological goal) actually corresponds to an entire movement trajectory (apart from starting position). As a result, the most behaviorally meaningful part of movement is not separately identified and therefore can’t be prioritized. In contrast, it supports models which make use of symbolic representations in the phonology. This is because symbolic representations can map onto a particular part of movement that relates most directly to the achievement of a (symbolic) phonological goal, and can therefore be prioritized for accuracy.

### The Observation of Less Timing Variability at Movement Endpoints Than at Other Parts of Movement Challenges Onset-Based Movement Coordination

Findings of greater temporal precision at endpoints compared to other parts of movement also provide evidence for the nature of movement coordination. It suggests that movement coordination is based on goal-related part(s) of movement (often the endpoint), and requires a way to ensure timing accuracy at the goal-related parts of movement. Additional evidence for goal-related, endpoint-based coordination in non-speech activity can be found in Gentner et al. (1980), Bootsma and van Wieringen (1990), Kazennikov et al. (1994), Haggard and Wing (1998), Craig et al. (2005), and Katsumata and Russell (2012); endpoint-based coordination and its implications for speech timing models are discussed at length in Turk and Shattuck-Hufnagel (2020).

### Summary of Evidence

To summarize, the above evidence suggests that (1) speakers represent and specify surface duration goals for intervals, (2) in specifying surface durations, speakers make use of general-purpose timekeeping mechanisms, and (3) speakers separately represent, and prioritize for timing coordination accuracy, the most behaviorally meaningful parts of movement. This evidence is inconsistent with approaches to speech production in which (1) surface timing characteristics are only emergent and not represented, (2) timing units do not relate straightforwardly to solar time, and (3) phonological representations define the timing of all parts of a movement trajectory. Instead, the evidence presented above motivates speech production models that make use of (1) general-purpose timekeeping mechanisms to represent and specify surface durations, and (2) have a way of representing behaviorally meaningful parts of movement separately from other parts of movement, so that they can prioritized for timing accuracy, and be coordinated with other events. In the following sections, we discuss why AP/TD, the model with the most comprehensive account of articulatory timing behavior, is challenged by these phenomena, and why these phenomena support a model based on symbolic representations and phonology-extrinsic timing, with three components: (1) Phonological Planning, (2) Phonetic Planning, and (3) Motor-Sensory Implementation.

## Discussion of AP/TD as the Most Comprehensive, Best-Worked Out Model of Timing and Why it is Nevertheless Challenged by These Findings

Articulatory Phonology in the Task Dynamics framework is currently the model with the most comprehensive coverage of timing effects in speech, including smooth, single-peaked velocity profiles, durations of gestural (constriction-forming) movements, coordination, boundary- and prominence-related lengthening, poly-sub-constituent shortening, and rate of speech. Key features of its approach to timing include (1) the use of spatio-temporal phonological representations, called gestures, as units of lexical contrast and phonological equivalence, (2) phonology-intrinsic timekeeping and gestural activation adjustment mechanisms to account for systematic contextual variability, (3) surface timing characteristics that emerge from the phonology without any representation of their durations in solar timing units. Its use of spatio-temporal phonological representations as units of lexical contrast and phonological equivalence, as well its commitment to emergent timing, are both motivated by the desire to avoid translation between phonological data structures to different data structures in phonetics (Fowler et al., 1980). That is, it is motivated by the desire to avoid having a phonetic planning component that is separate from the phonology. A substantial challenge of this approach is how to account for systematic variability in the production of members of the same phonological category, while maintaining their phonological equivalence. Its solution to this challenge involves (1) a definition of the gesture which allows for different gestural starting positions; (2) phonology-intrinsic mechanisms for adjusting the interval of time that a gesture is active (its gestural activation interval); and (3) mechanisms for controlling patterns of gestural overlap, and articulatory activity that it governs. Together, these mechanisms give rise to surface contextual variability without altering the defining characteristics of each gesture, i.e., its equation of motion and coefficient values (apart from starting position).

In this section, we lay out AP/TD’s approach to this challenge in more detail. Gestures are modeled by a second order mass-spring equation of oscillatory motion, critically damped so that the mass approximates a target position but doesn’t oscillate. Target and stiffness coefficient values vary across gestural categories (target values differ for each gesture; stiffness values differ for consonants vs. vowels), whereas damping and mass coefficients are the same for all gestures (mass is set to 1, damping has a value that ensures critical damping). Gestural starting position is determined by previous context. How long each gesture is active (the gestural activation interval) is controlled by a system of coupled limit-cycle oscillators, i.e., gestural planning + syllable, foot and phrase oscillators (Saltzman et al., 2008, but see Tilsen, 2013, 2016, 2018, for a different approach). Because the gestural activation interval is defined as a proportion of a gestural planning oscillator cycle, the oscillation rate of the gestural planning + suprasegmental oscillator ensemble determines the duration of the gestural activation interval. A default oscillation rate gives rise to activation intervals which are long enough for gestures to approximate their targets. Longer intervals at phrase edges and in prominent positions are achieved via a mechanism which slows the AP/TD “clock” in these positions without adding timing units (see Byrd and Saltzman, 2003 for an early Pi gesture proposal; Saltzman et al., 2008 for a later Mu_*T*_ proposal which slows the gestural planning + suprasegmental oscillator ensemble oscillation rate); shorter intervals for, e.g., faster rates of speech, are achieved by speeding up the planning + suprasegmental oscillator “clock” (Saltzman et al., 2008), which may result in undershoot of the stored gestural target.

In this model, surface timing characteristics are the emergent output of fixed gestural characteristics (e.g., the time it takes for the gestural mass-spring system to approximate its target position), as well as utterance-specific gestural activation interval specifications (determined by the oscillation frequency of the planning + suprasegmental oscillator ensemble and the shape of the activation interval on- and off-ramps). Articulatory and acoustic surface timing characteristics (as measurable in solar timing units) emerge from this system without the involvement of any phonology-extrinsic, general-purpose timekeeping mechanisms that operate with such units. Because the planning + suprasegmental oscillator “clock” frequency is changed in particular phrasal positions and for different overall speech rates, there is no straightforward correspondence between planning + suprasegmental oscillator “clock” timing units and solar timing units.

Despite the general success of this model in accounting for timing effects in speech, it is challenged by the findings presented in previous sections. We will briefly review those challenges in light of the characteristics of AP/TD described just above.

### Constraints on Lengthening Due to Phrasal Prosody

Constraints on lengthening phonemically short vowels in prosodic contexts where lengthening occurs are difficult to explain in AP/TD. On the assumption that the lexical difference between short and long vowels in AP/TD is a difference in phonological representation, e.g., of one vs. two gestures, or of a gesture associated with one vs. two moras, both phonemically short and long vowels could be lengthened by the same amount and the lexical distinction would be maintained. But, that is not what is observed. Instead, less lengthening is found on the short vowel. This can only be accomplished in AP/TD by an *ad hoc* imposition of a smaller amount of lengthening on the short vowel, e.g., via a Pi/Mu_*T*_ gesture with a smaller height for phonemically short vowels, or by proposing a Pi/Mu_*T*_ phasing solution, in which (1) a Pi/Mu_*T*_ gesture is aligned to the onset of the final syllable, (2) the Pi/Mu_*T*_ gesture is of fixed duration, and (3) the Pi/Mu_*T*_ gesture activation increases over time. However, although AP/TD provides these possible mechanisms for *implementing* different degrees of lengthening on phonemically short vs. long vowels, it provides no *explanation* of the phenomenon. That is, AP/TD provides no explanation for why phonemically short vowels should have Pi/Mu_*T*_ gestures with shorter heights associated with them, or why a Pi/Mu_*T*_ gesture would need to be phased with respect to the onset of a final syllable in Finnish, but not in other languages. For example, in non-quantity languages with reduced vs. full vowels, the proportional magnitude of boundary-related lengthening is not constrained for short duration, reduced vowels (e.g., the unstressed vowel in *Thomas*) as compared to longer duration full-vowels (e.g., the second syllable vowel in *Brookline*, Turk and Shattuck-Hufnagel, 2007). This suggests alignment of a Pi/Mu_*T*_ gesture with the end of a word (Byrd and Saltzman, 2003), rather than with the onset of the final syllable.

In contrast to AP/TD, which has difficulty explaining the constraint on final lengthening on phonemically short vowels in quantity languages, theories which allow for the representation of surface durations provide a possible explanation. That is, smaller amounts of lengthening on phonemically short vowels can be explained if there is a constraint that preserves the surface duration distinction between phonemically short and long vowels.

To put it another way, if vowels of different quantities had the same phonological representation, the constraint on prosodic lengthening for short (and medium) vowels could be expressed as a constraint on the degree of AP/TD “clock” slowing. But, in this case, where the two types of vowels had the same phonological representation (i.e., the same number and type of gesture), there would be no way to express the lexical contrast. Instead, because AP/TD differentiates phonological categories with gestures, we assume that the phonological contrast between these different types of vowels is expressed in the lexicon as one vs. two or more gestures or perhaps as a single gesture associated with one vs. two moras. As a result, the surface durations of these vowels is due to a combination of (1) the number of AP/TD timing units in their gestural activation intervals (determined by the number of gestures or the number of moras), and (2) the degree of clock slowing (determined by the Pi or Mu_*T*_ gesture). In this type of system, there is no way to account for the apparent surface duration constraint on the lengthening of contrastively short vowels, because this constraint relates to the emergent result of the interaction of two different AP/TD properties: (1) the number of AP/TD “clock” timing units in the activation interval and (2) the degree of clock slowing, which together result in surface duration in solar time. AP/TD can refer to each of these quantities, but has no way of representing the fact that they both affect surface duration, that is, it has no way of relating their equivalent effects on a desired surface duration. AP/TD therefore has no explanation for different degrees of clock slowing on phonemically short vs. long vowels, because the explanation has to do with the maintenance of a surface duration distinction.

In sum, while the AP/TD phonology-intrinsic “clock”-slowing Pi- and Mu_*T*_-gestures might provide a mechanism to specify different degrees of phrase-final- or phrasal-accent-related lengthening for contrastively short (or medium) vs. long vowels, AP/TD has no representation of the surface duration outcomes of such activation interval adjustments. Consequently, it does not predict that a difference in lengthening degree for phonemically short vs. long vowels should occur, and does not offer an explanation for *why* contrastively short vowels are lengthened less, nor for the degree of lengthening these contrastively short vowels exhibit. Furthermore, adding a representation of the surface duration outcome would not be desirable in this framework, because this would involve a “translation” of phonology-intrinsic time into (phonetic) surface durations, something that the authors of the framework (and its antecedents) have tried to avoid (Fowler et al., 1980).

### Different Strategies for Manipulating Durations in, e.g., Rate of Speech, Boundary-Related Lengthening, and Quantity

This evidence suggests the equivalence between different temporal and spatio-temporal strategies that accomplish the same surface duration goal. It is challenging to account for in AP/TD for two reasons: (1) AP/TD doesn’t have a representation of surface duration goals, and (2) AP/TD doesn’t make a distinction between goals and how the goals are achieved. In this model, there are several different mechanisms that result in longer surface durations, e.g., differences in gestural stiffness, slowing gestural planning oscillators for longer gestural activation intervals, and adding gestures. However, because the model cannot refer to surface durations, the explanatory fact that these mechanisms all have a similar surface duration result is not captured in the model. Furthermore, in AP/TD, spatial and temporal aspects of movement are not independent: both are determined by the same phonological plan. Thus, it is difficult to account for behavior in which a speaker obtains the same temporal result with different spatial paths. Put another way, it is difficult for this model to account for the equivalence of rates, of quantities, and of lengthening (e.g., in final position) when these are achieved in different temporal and/or spatial manners, because this model doesn’t allow the specification of temporal goals as distinct from the way they are achieved. That is, in AP/TD this equivalence can only arise by chance, because achieving the same surface duration pattern result can’t be specified as the goal of the speaker.

### More Timing Variability for Longer Duration Intervals

Findings of greater timing variability in phrase-final and in prominent positions are inconsistent with AP/TD’s account of boundary-related and prominence-related lengthening, with its lack of surface durations, and with its lack of general-purpose timekeeping mechanisms. To see why this is so, first consider the details of how timing is adjusted in this model. In AP/TD, longer surface durations in phrase-final and prominent positions result from Pi or Mu_*T*_ adjustments, which stretch gestural activation intervals in these positions. In recent versions of the model (Saltzman et al., 2008) this stretching is done by slowing the phonology-specific “clock,” which is accomplished by slowing the oscillation frequency of an ensemble of gestural planning + suprasegmental oscillators. Because the duration of each gestural activation interval corresponds to a proportion of a planning oscillator period, slowing the gestural planning + suprasegmental ensemble of oscillators stretches the activation interval. Because this clock-slowing mechanism slows the phonology-specific clock without adding any extra timing units, intervals in phrase-final and prominent positions are not actually longer in phonology-specific clock time, even though they are longer in surface time.

These operational details and their implications are significant because they highlight the difficulty of accounting for greater timing variability for intervals that are longer in surface time but not in the number of phonology-specific timing units. That is, greater timing variability observed for longer surface duration intervals is straightforward to account for in a model where timing variability correlates with the number of timing units. AP/TD can use this type of account for the greater timing variability observed for longer duration phonemically long vowels as compared to shorter duration phonemically short vowels (cf. Figures 1 and 2 for examples of this variability pattern in N. Finnish and Dinka). This is because longer durations for phonemically long vowels correspond to greater numbers of phonology-specific timing units, e.g., phonemically long vowels are assumed to be composed of two gestures (or are potentially associated with two moras), with corresponding longer gestural activation intervals. However, AP/TD does not have an account for the greater timing variability for movements in phrase-final or phrasally prominent positions, since gestural activation intervals in these positions have the same number of AP/TD timing units as corresponding gestural activation intervals in phrase-medial, or non-prominent positions. This is because longer surface durations in these positions are due to AP/TD phonology-specific clock-slowing, rather than to a greater number of AP/TD phonology-specific clock units.

The number of phonology-specific timing units therefore is not a quantity that can be used to account for temporal variability within AP/TD. Neither is the degree of lengthening (i.e., of phonology-specific clock slowing as implemented through the height of a Pi or Mu_*T*_ gesture): Adding noise in proportion to Pi or Mu_*T*_ height might add timing variability of surface durations of long vowels, but would not explain the fact that phrase-medial unstressed vowels that are not accompanied by Pi or Mu_*T*_ gesture lengthening also show timing variability.

The findings instead argue for the representation of surface duration as a quantity, which is absent from AP/TD. In addition, AP/TD’s reliance on phonology-specific timekeeping mechanisms provides no account of the similarity in timing variability behavior between speech and non-speech activity. This finding is more consistent with the use of noisy, general-purpose timekeeping mechanisms in both domains (e.g., Schöner, 2002 and many others). That is, in AP/TD, the fact that general-purpose timekeepers governing other motor behaviors, and the proposed phonology-intrinsic timekeeper, share the characteristic of greater variability for longer intervals goes unexplained.

### The Observation of Less Timing Variability at Goal-Related Parts of Movement

#### A Challenge to Spatio-Temporal Phonological Representations

These data are problematic for AP/TD because they suggest that actors are able to separately represent, and differentially prioritize, the timing of different components of movement, e.g., endpoints over other parts of movement, such as movement onset. These findings are difficult to explain in models such as AP/TD, where a phonological representation takes the form of equations that, together with gestural activation, define the full trajectory of a gestural movement (as well as the trajectories of the individual movements of the articulators that form the gesture), once starting position has been specified. Thus, it is not possible to represent either the spatial or temporal aspects of one part of a movement (e.g., the endpoint) separately from the other parts of the movement trajectory. As a result, it is not possible to prioritize greater timing accuracy for different parts of a movement separately.

Note that the fact that the movement target is a parameter of movement in AP/TD does not mean that the target can be singled out as a part of movement that is independent of other parts. This is because the movement target parameter value, along with values for starting position, spring stiffness, mass, and damping parameters, affect the entire trajectory of movement, defined by the mass-spring equation, its activation, and overlap with other gestures.

It is important to note also, that even if a part of movement could be identified in this type of framework, there is nothing in the model that would predict different timing variability for a particular part of movement. For example, the timing of movement onset can be identified in this model as the onset of gestural activation. However, because the timing of all parts of movement is defined by the same equation of motion, and its gestural activation interval, there is no available mechanism to differentially prioritize any particular part of movement over another part for timing accuracy. And perhaps most importantly, because the entire movement trajectory (minus its starting position) represents the goal of movement, there is no principled reason for any part of movement to be prioritized for timing accuracy over any other part.

In sum, the evidence presents two challenges: The first, that individual parts of movement cannot be identified, is partially addressed in that movement onsets can be identified with the onset of gestural activation; however but crucially, movement endpoints cannot. The second challenge, that some parts of movement are more accurately timed than others, cannot be met because the equation of motion that describes the phonological representation defines the spatial and temporal properties of the entire gesture, apart from its starting position. Thus no part of it can vary independently of any other.

#### A Challenge to Onset-Based Movement Coordination

These findings also suggest that coordination patterns can be based on the part of movement most closely related to the phonological goal, often the movement endpoint, instead of the movement onset, as currently implemented in AP/TD. Whereas the movement onset corresponds to the onset of gestural activation, the movement endpoint is much more difficult to identify in this framework. This is because the time of gestural target approximation is determined primarily from properties of the gestural mass-spring, point-attractor oscillator, and only relates straightforwardly to the duration of an activation interval at a default speaking rate. That is, at a default speaking rate, gestural activation interval durations correspond to the planning oscillator phase proportion that gives each gesture enough time to approximate its target. However, when activation intervals are adjusted for different speaking rates, or for prominent, or boundary-adjacent position, the time of gestural approximation will no longer correspond to a fixed phase of a planning oscillator (e.g., the end of gestural activation). Specifically, if the gestural activation interval is longer than the time it takes for the gesture to approximate its target (because the planning oscillator system has been slowed, e.g., in boundary-adjacent position), then the end of gestural activation will not correspond to the point of target approximation, and will occur later. Put another way, the time of gestural target approximation cannot be identified as a particular phase of a gestural planning oscillator at speaking rates different from the default, and in prosodic contexts (e.g., boundary-adjacent positions, and phrasally prominent positions) where gestural activation intervals have been stretched. This is because in these contexts, gestural planning oscillator frequency (which determines how long gestures are active), is independent of the natural frequency of gestures themselves (which is invariant and determined by properties of the gestural point-attractor mass-spring system Byrd and Saltzman, 2003; Saltzman et al., 2008). Because it is the natural frequency of each gesture that is primarily responsible for the timing of target approximation (Saltzman and Munhall, 1989), it is challenging to identify a movement endpoint (or the time of target approximation) in the current AP/TD framework^11^. We note that tying gestural movement timing more closely to gestural activation timing (so that endpoints could be identified with a particular phase of a planning oscillator cycle) would present additional problems, e.g., overly long movement durations in contexts where gestural activation must be long (e.g., in phrase-final positions or at slow speaking rates). For example, if a singer is asked to sing a single syllable /bɑ/ for a long period of time on a single note, s/he will typically move from a bilabial target to the vowel target relatively quickly, and then prolong the /ɑ/ vowel by maintaining the oral tract in a quasi-“steady state,” target position for the vowel. If the movement toward the vowel target is slowed down in proportion to the duration of the note, the speaker would end up producing what might sound like a continuum of vowel-like sounds between the release of [b] and the target for [ɑ].

The findings presented above thus challenge the chosen architecture of AP/TD, with its spatio-temporal representations, lack of separation between Phonological and Phonetic Planning Components, phonology-intrinsic timing, and emergent (rather than explicitly represented) surface phonetic characteristics. We suggest that providing accounts of the phenomena described in earlier sections of the paper may be difficult without sacrificing some of the core principles of this theory’s current implementation. For example, accounting for less timing variability at a part of movement most closely related to a goal challenges the core principle of an integrated phonology-phonetics, in which the phonological representation both serves as the goal of movement, and provides instructions for implementing the movement. And the evidence for the representation of surface durations may be difficult to accommodate in such a system, without sacrificing spatio-temporal phonological representations and without having to translate from data structures in phonology to different data structures on the surface (in phonetics). The current system of adjusting gestural activation intervals in different contexts, while preserving the invariance of gestural representations, allowed the theory (1) to account for the phonological equivalence of the same gestures in different contexts, (2) to account for different (emergent) surface behavior of these gestures in different contexts, (3) to do both of these things without a separate Phonetic Planning Component that would provide translation from qualitatively different phonological representations to quantitatively specified surface phonetic forms. However, the findings presented in this paper suggest the need for just such a process, i.e., it suggests that the surface duration results of the adjustment processes are represented, and require translation from the data structures in phonology to those in phonetics.

## Why the Findings Point Toward a 3-Component Model Based on Symbolic Phonological Representations and Phonology-Extrinsic Timing

In this section, we argue that the findings presented above motivate the consideration of models of speech production with three components: (1) Phonological Planning, (2) Phonetic Planning, and (3) Motor-Sensory Implementation. Those findings provide a number of lines of evidence that support an approach of this kind, which is based on phonology-extrinsic timing and symbolic phonological representations. First, several findings suggested that surface durations are represented in the minds of speakers, and furthermore that these durations are specified through the use of non-speech-specific, general-purpose mechanisms, in solar timing units. Because this evidence supports mechanisms for quantitative specification that are extrinsic to the phonology, it can easily be accommodated in a model of speech production in which quantitative specification occurs in a phonetic component that is separate from the symbol-based phonological plan (which does not contain specific spectral, spatial or temporal information) that the speaker develops for a particular utterance.

Further support for a model of speech motor control that has a separation between Phonological and Phonetic Planning Components is provided by findings of greater temporal accuracy at behaviorally meaningful parts of movement. These findings also motivate a third, Motor-Sensory Implementation Component that is separate from the two planning components, and is used for tracking and adjusting movements once they have begun. That is, the findings presented earlier can be explained if (1) a particular part of movement (e.g., the endpoint or possibly constriction release) is identified as “behaviorally meaningful,” i.e., most closely related to the goals specified in the symbolic phonological plan (which is developed during the operation of a Phonological Planning Component) that the speaker is trying to signal, and (2) other aspects of the movement (specified during the operation of a Phonetic Planning Component) are organized in the service of reaching the behaviorally meaningful (and thus high-priority) part of movement at the right time, and with appropriate temporal and spatial accuracy. As a result, parts of movement that are less directly related to the goal are less likely to be corrected and adjusted during the operation of the Motor-Sensory Implementation Component, because their accuracy is less critical, as long as the goal-related part can be reached on time (cf. Todorov and Jordan, 2002, 2003, Minimal Intervention Principle). Instead, the resources for tracking and adjusting are focused on the aspects of a movement that are most closely related to the goal of producing a planned set of acoustic cues, e.g., its endpoint, or release from constriction.

To put this another way, in a three-component model that separates the phonological goal (as an abstract, symbolic, phonological element in an appropriate utterance-specific context) from the manner of carrying out the goal (as a quantitative phonetic specification that includes movement duration in solar timing units, e.g., ms)^12^, it is possible to relate the symbolic phonological goal to the part(s) of articulatory movement that are most closely related to achieving that phonological goal. Because those parts of movement have a separate representation from other parts, it is possible to prioritize them for temporal coordination, and for more accurate production in a motor-sensory implementation component. This is precisely what appears to be required by the distribution of timing accuracy across a movement. The identification of this part of a movement with the phonological goal of movement provides a rationale for why that particular part of movement should be given higher priority with regard to timing and/or spatial accuracy. In a three-component approach, the Motor-Sensory Implementation Component, which tracks timing and position relative to the endpoint (presumably based on prediction from an efference copy of the motor commands as well as on sensory information), is required to provide adjustments to the movements as they unfold, in order to ensure that the prioritized endpoint is reached at an appropriate time.

Several models in the literature are 3-component models, with separate Phonological Planning, Phonetic Planning, and Motor-Sensory-Implementation Components, and make use of surface durations, specified in solar timing units, and of phonology-extrinsic general-purpose timekeeping mechanisms (e.g., Fujimura, 1992 et seq., Guenther, 2016). These models are therefore promising, because they are compatible with many of the findings detailed above. However, in spite of their use of symbolic phonological representations, in some cases these models have identified the goals of movement as entire movements (Fujimura, 1992 et seq.), or as spectro-temporal trajectories (Guenther, 2016), as opposed to identifying the goals as particular parts of movement. These modeling decisions are at odds with findings of greater timing accuracy at goal-related parts of movement. We suggest that if these models were modified to map phonological goals onto particular parts of movement, they would be compatible with the findings presented here. Lee’s (1998) tau-coupling theory provides a way to account for less timing variability at goal-related parts of movement, because in that theory, movements are guaranteed to reach their goals at a particular time even if the timing of movement onset is variable. Lee’s theory also provides a principled way to account for the time-course of movement (and resulting velocity profile shapes).

One challenge for any model of speech production, including 3-component models that use symbolic phonological representations and phonology-extrinsic timekeeping mechanisms, is to account for the systematic influence of a wide range of factors on timing patterns in speech. Optimal Control Theory approaches are promising in this regard, because they provide a way to balance the costs of not achieving movement goals (e.g., signaling phonemic contrast, in ways that are appropriate in particular prosodic positions, using a particular style, at an appropriate rate, etc.), with movement costs. See e.g., Šimko and Cummins (2010, 2011), Windmann et al. (2015), and Windmann (2016) for examples of ways that Optimal Control Theory approaches can be used to predict systematic timing patterns in speech. However, if these approaches are to be taken as theories of speech production, they present their own challenges; for example, they require extensive computation every time an utterance is planned.

In summary, evidence from the timing literature suggests that models of speech production based on symbolic representations and phonology-extrinsic timing are worth developing as alternatives to the currently dominant AP/TD approach, in spite of their computational challenges. See Turk and Shattuck-Hufnagel (2020) for a sketch of a specific proposal for how this might be done.

## Author Contributions

AT synthesized and interpreted the evidence from the timing literature. AT and SS-H wrote the manuscript.

## Conflict of Interest

The authors declare that the research was conducted in the absence of any commercial or financial relationships that could be construed as a potential conflict of interest.

## Footnotes

1 The surface articulatory trajectories controlled by a given gesture are determined by the gesture itself, as well as context, i.e., gestural starting position, overlap with other gestures, prosodic context, and speaking rate.

2 Like Gafos (2006) and Gafos and Beňuš (2006), Flemming’s (2001) approach involves multiple, competing target specifications, but Flemming (2001) doesn’t explicitly model speech articulation.

3 An additional difference between the spatio-temporal vs. symbolic approaches is that in spatio-temporal approaches, a phonological representation (gesture) is defined by an equation of motion with a lexically specified and fixed gestural target coefficient. And each gesture controls a fixed set of articulators (a coordinative structure) for the production of an articulatory constriction, although the relative contribution of each articulator in producing a gesture can vary according to context. In contrast, in symbolic approaches, the constriction targets, and even the sets of articulators, used for the production of different tokens of the same symbolic phonological category can vary. For example, the symbolic feature [+labial] can be produced with a labiodental constriction target (for [v]) or with a labial constriction target (for [b]), and in British varieties of English the phoneme /t/ can be produced with and without involvement of the tongue tip, as in aspirated [t^*h*^] and glottal stop variants [ʔ] (Heyward et al., 2014, see additional examples and discussion in Turk and Shattuck-Hufnagel, 2020).

4 Embodied Task Dynamics was not intended to be a theory of online speech production, but rather was deverloped to explain coordination patterns.

5 Speech-ready stiffness is analogous to the stiffness of the neutral attractor in the AP/TD framework, except that speech-ready dynamics is always turned on, even when gestures are active, and the speech-ready stiffness of individual articulators can be manipulated according to requirements for higher precision (Šimko, 2009). The speech-ready position is assumed to be “an average constellation with regard to the entire set of mastered gestures” (Šimko et al., 2014, p. 133).

6 Windmann et al. (2015) and Windmann (2016) show how this same general approach, i.e., minimizing costs of effort, (mis)-parsing and time, can be used to model durational effects of prominence (phrasal prominence, lexical prominence, and their interaction) and polysyllabic shortening, as well as interactions with speaking rate, as measured from the acoustic signal.

7 The “half-long vowel” is a phonemically short vowel whose phrase-medial duration is intermediate between that of a non-word-final phonemically short vowel and that of the long vowel (VV).

8 It is often the case that the part of movement most closely related to the goal in speech is the movement endpoint. For example, the endpoint of lip protrusion is most closely to the goal for /u/, but for geminate consonants followed by a vowel, the timing of the beginning of the release movement toward the following vowel may be the most relevant for signaling the geminate status of the consonant.

9 In cases where movements are of different distances, additional variability in the timing of movement onsets might be expected, since Fitts’ (1954) law dictates longer duration movements for longer distances (in spite of higher velocities for these movements) as long as accuracy or target width remains constant.

10 Although they do not report magnitudes of variability, e.g., in standard deviations, the difference in variability is clear on visual inspection of the figure.

11 Researchers working in the AP framework, e.g., Browman and Goldstein (1989, 1992), Davidson (2006), describe some patterns of observed data in terms of coordination patterns based on parts of movement other than the onset (e.g., target and release); however, they do not provide explicit mechanisms for identifying these parts of movement so they can be coordinated.

12 Note that phonological representations in our proposal can include discrete, relational and/or e.g. binary quantity representations, but not gradient, scalar representations.

## References

[B1] Abbs J. H. (1973). The influence of the gamma motor system on jaw movements during speech: a theoretical framework and some preliminary observations. *J. Speech Hear. Res.* 16 175–200. 10.1044/jshr.1602.175 4269271

[B2] Beňuš Š. Šimko J. (2014). Emergence of prosodic boundary: continuous effects of temporal affordance on inter-gestural timing. *J. Phonetics* 44 110–129. 10.1016/j.wocn.2013.12.005

[B3] Berry J. (2011). Speaking rate effects on normal aspects of articulation: outcomes and issues. *Perspect. Speech Sci. Orofacial Disord.* 21 15–26.

[B4] Billon M. Semjen A. Stelmach G. E. (1996). The timing effects of accent production in periodic finger-tapping sequences. *J. Mot. Behav.* 28 198–210. 10.1080/00222895.1996.9941745 12529203

[B5] Birkholz P. Kröger B. J. Neuschaefer-Rube C. (2011). Model-based reproduction of articulatory trajectories for consonant–vowel sequences. *IEEE Trans. Audio Speech Lang, Process*, 19 1422–1433. 10.1109/tasl.2010.2091632

[B6] Bootsma R. J. van Wieringen P. C. (1990). Timing an attacking forehand drive in table tennis. *J. Exp. Psychol.* 16 21–29. 10.1037//0096-1523.16.1.21

[B7] Boyce S. E. Krakow R. A. Bell-Berti F. Gelfer C. E. (1990). Converging sources of evidence for dissecting articulatory movements into core gestures. *J. Phonetics* 18 173–188. 10.1016/s0095-4470(19)30400-0

[B8] Browman C. P. Goldstein L. (1985). “Dynamic modeling of phonetic structure,” in *Phonetic Linguistics*, ed. Fromkin V. A., (New York, NY: Academic Press), 35–53.

[B9] Browman C. P. Goldstein L. (1989). Articulatory gestures as phonological units. *Phonology* 6 201–251. 10.1017/s0952675700001019

[B10] Browman C. P. Goldstein L. (1992). Articulatory phonology: an overview. *Phonetica* 49 155–180. 10.1159/000261913 1488456

[B11] Byrd D. Saltzman E. (1998). Intragestural dynamics of multiple prosodic boundaries. *J. Phonetics* 26 173–199. 10.1006/jpho.1998.0071

[B12] Byrd D. Saltzman E. (2003). The elastic phrase: modeling the dynamics of boundary-adjacent lengthening. *J. Phonetics* 31 149–180. 10.1016/s0095-4470(02)00085-2

[B13] Byrd D. Tan C. C. (1996). Saying consonant clusters quickly. *J. Phonetics* 24 263–282. 10.1006/jpho.1996.0014

[B14] Chen Y. (2006). Durational adjustment under corrective focus in Standard Chinese. *J. Phonetics* 34 176–201. 10.1016/j.wocn.2005.05.002

[B15] Craig C. Pepping G.-J. Grealy M. (2005). Intercepting beats in predesignated target zones. *Exp. Brain Res.* 165 490–504. 10.1007/s00221-005-2322-x 15912367

[B16] Davidson L. (2006). Phonotactics and articulatory coordination interact in phonology: evidence from nonnative production. *Cogn. Sci.* 30 837–862. 10.1207/s15516709cog0000_73 21702839

[B17] Edwards J. Beckman M. E. Fletcher J. (1991). The articulatory kinematics of final lengthening. *J. Acoust. Soc. Am.* 89 369–382. 10.1121/1.400674 2002175

[B18] Engstrand O. (1988). Articulatory correlates of stress and speaking rate in Swedish CV utterances. *J. Acoust. Soc. Am.* 88 1863–1875. 10.1121/1.396522 3403802

[B19] Fitts P. M. (1954). The information capacity of the human motor system in controlling the amplitude of movement. *J. Exp. Psychol.* 47 381–391. 10.1037/h0055392 13174710

[B20] Flemming E. (2001). Scalar and categorical phenomena in a unified model of phonetics and phonology. *Phonology* 18 7–44. 10.1017/s0952675701004006

[B21] Fletcher J. (1987). Some micro and macro effects of tempo change on timing in French. *Linguistics* 25 951–967.

[B22] Fowler C. A. Rubin P. Remez R. E. Turvey M. T. (1980). “Implications for speech production of a general theory of action,” in *Language Production*, ed. Butterworth B., (New York: Academic Press), 373–420.

[B23] Fujimura O. (1992). Phonology and phonetics-A syllable-based model of articulatory organization. *J. Acoust. Soc. Japan (E)* 13 39–48. 10.1250/ast.13.39

[B24] Fujimura O. (1994). “C/D model: a computational model of phonetic implementation,” in *DIMACS Series in Discrete Mathematics and Theoretical Computer Science*, ed. Ristad E. S., Vol. 17, 1–20.

[B25] Fujimura O. (2002). Temporal organization of speech utterance: a C/D model perspective. *Cadernos de Estudos Lingüísticos* 43 9–36. 10.20396/cel.v43i0.8637147

[B26] Gafos A. I. (2006). “Dynamics in grammar: comment on Ladd and Ernestus & Baayen,” in *Laboratory Phonology 8*, eds Goldstein L. Whalen D. H. Best C. T., (Berlin: Walter de Gruyte), 51–80.

[B27] Gafos A. I. Beňuš S. (2006). Dynamics of phonological cognition. *Cogn. Sci.* 30 905–943. 10.1207/s15516709cog0000_80 21702841

[B28] Gafos A. I. Charlow S. Shaw J. A. Hoole P. (2014). Stochastic time analysis of syllable-referential intervals and simplex onsets. *J. Phonetics* 44 152–166. 10.1016/j.wocn.2013.11.007

[B29] Gafos A. I. Roeser J. Sotiropoulou S. Hoole P. Zeroual C. (2019). Structure in mind, structure in vocal tract. *Nat. Lang. Linguist. Theor.* 10.1007/s11049-019-09445-y

[B30] Gallistel C. R. (1999). Can response timing be explained by a decay process? *J. Exp. Anal. Behav.* 71 264–271. 10.1901/jeab.1999.71-264 10366312 PMC1284707

[B31] Gallistel C. R. Gibbon J. (2000). Time, rate, and conditioning. *Psychol. Rev.* 107 289–344. 10.1037//0033-295x.107.2.289 10789198

[B32] Gentner D. R. Grudin J. Conway E. (1980). “Finger movements in transcription typing,” in *Center for Human Information Processing*, Technical Report 8001 (San Diego: University of California), 1–8.

[B33] Getty D. J. (1975). Discrimination of short temporal intervals: a comparison of two models. *Percept. Psychophys.* 18 1–8. 10.3758/bf03199358

[B34] Gibbon J. (1977). Scalar expectancy theory and Weber’s law in animal timing. *Psychol. Rev.* 84 279–325. 10.1037/0033-295x.84.3.279

[B35] Goldstein L. Nam H. Saltzman E. Chitoran I. (2009). “Coupled oscillator planning model of speech timing and syllable structure,” in *Frontiers in Phonetics and Speech Science*, eds Fant G. Fujisaki H. Shen J., (Beijing: The Commercial Press), 239–250.

[B36] Goozée J. V. Lapointe L. L. Murdoch B. E. (2003). Effects of speaking rate on EMA-derived lingual kinematics: a preliminary investigation. *Clin. Linguist. Phonetics* 17 375–381. 10.1080/0269920031000079967 12945613

[B37] Green J. T. Ivry R. B. Woodruff-Pak D. S. (1999). Timing in eyeblink classical conditioning and timed-interval tapping. *Psychol. Sci.* 10 19–23. 10.1111/1467-9280.00100 9640581

[B38] Guenther F. H. (1995). Speech sound acquisition, coarticulation, and rate effects in a neural-network model of speech production. *Psychol. Rev.* 102 594–621. 10.1037//0033-295x.102.3.594 7624456

[B39] Guenther F. H. (2016). *Neural Control of Speech.* Cambridge, MA: The MIT Press.

[B40] Guenther F. H. Micci Barreca D. (1997). “Neural models for flexible control of redundant systems,” in *Self-Organization, Computational Maps, and Motor Control*, eds Morasso P. Sanguineti V., (Amsterdam: Elsevier Science B.V), 383–421. 10.1016/s0166-4115(97)80014-3

[B41] Haggard P. Wing A. (1998). Coordination of hand aperture with the spatial path of hand transport. *Exp. Brain Res.* 118 286–292. 10.1007/s002210050283 9547099

[B42] Harris C. M. Wolpert D. M. (1998). Signal-dependent noise determines motor planning. *Nature* 394 780–784. 10.1038/29528 9723616

[B43] Henke W. L. (1966). *Dynamic Articulatory Model of Speech Production Using Computer Simulation.* PhD dissertation, Massachusetts Institute of Technology, Cambridge, MA.

[B44] Hertrich I. Ackermann H. (1997). Articulatory control of phonological vowel length contrasts: kinematic analysis of labial gestures. *J. Acoust. Soc. Am.* 102 523–536. 10.1121/1.419725 9228815

[B45] Heyward J. Turk A. Geng C. (2014). “Does /t/ produced as [ɑ] involve tongue tip raising? Articulatory evidence for the nature of phonological representations,” *Paper Presented at the 14th Conference on Laboratory Phonology*, Tokyo.

[B46] Houde J. F. Nagarajan S. S. (2011). Speech production as state feedback control. *Front. Hum. Neurosci.* 5:82. 10.3389/fnhum.2011.00082 22046152 PMC3200525

[B47] Ivry R. B. Hazeltine R. E. (1995). Perception and production of temporal intervals across a range of durations - Evidence for a common timing mechanism. *J. Exp. Psychol. Hum. Percept. Perform.* 21 3–18. 10.1037/0096-1523.21.1.3 7707031

[B48] Jones L. A. Wearden J. H. (2004). Double standards: memory loading in temporal reference memory. *Q. J. Exp. Psychol.* 57B 55–77. 10.1080/02724990344000088 14690849

[B49] Katsumata H. Russell D. M. (2012). Prospective versus predictive control in timing of hitting a falling ball. *Exp. Brain Res.* 216 499–514. 10.1007/s00221-011-2954-y 22120106

[B50] Kazennikov O. Wicki U. Corboz M. Hyland B. Palmeri A. Rouiller E. M. (1994). Temporal structure of a bimanual goal-directed movement sequence in monkeys. *Eur. J. Neurosci.* 6 203–210. 10.1111/j.1460-9568.1994.tb00262.x 8167842

[B51] Keating P. A. (1990). “The window model of coarticulation: articulatory evidence,” *Papers Presented in Laboratory Phonology I: Between the Grammar and Physics of Speech*, eds Kingston J. C. Beckman M. E., (Cambridge, MA: Cambridge University Press), 450–469.

[B52] Lacquaniti F. Maioli C. (1994). Coordinate transformations in the control of cat posture. *J. Neurophysiol.* 72 1496–1515. 10.1152/jn.1994.72.4.1496 7823082

[B53] Lee D. N. (1998). Guiding movement by coupling taus. *Ecol. Psychol.* 10 221–250. 10.1207/s15326969eco103

[B54] Lee D. N. (2009). Lee’s 1976 paper. *Perception* 38 837–858. 19806967 10.1068/pmklee

[B55] Lefkowitz L. M. (2017). *Maxent Harmonic Grammars and Phonetic Duration.* Ph.D. thesis, University of California, Los Angeles, CA.

[B56] Leonard T. Cummins F. (2011). The temporal relation between beat gestures and speech. *Lang. Cogn. Process.* 26 1457–1471. 10.1080/01690965.2010.500218

[B57] Merchant H. Zarco W. Prado L. (2008). Do we have a common mechanism for measuring time in the hundreds of millisecond range? Evidence from multiple-interval timing tasks. *J. Neurophysiol.* 99 939–949. 10.1152/jn.01225.2007 18094101

[B58] Nakai S. Kunnari S. Turk A. Suomi K. Ylitalo R. (2009). Utterance-final lengthening and quantity in Northern Finnish. *J. Phonetics* 37 29–45. 10.1016/j.wocn.2008.08.002

[B59] Nakai S. Turk A. E. Suomi K. Granlund S. C. Ylitalo R. Kunnari S. (2012). Quantity constraints on the temporal implementation of phrasal prosody in Northern Finnish. *J. Phonetics* 40 796–807. 10.1016/j.wocn.2012.08.003

[B60] Nam H. Goldstein L. Saltzman E. (2010). “Self-organization of syllable structure: a coupled oscillator model,” in *Approaches to Phonological Complexity*, eds Pellegrino F. Marisco E. Chitoran I., (New York, NY: Mouton de Gruyter).

[B61] Nelson W. L. (1983). Physical principles of economies of skilled movements. *Biol. Cybernet.* 46 135–147. 10.1007/bf00339982 6838914

[B62] Ostry D. J. Munhall K. G. (1985). Control of rate and duration of speech movements. *J. Acoust. Soc. Am.* 77 640–648. 10.1121/1.391882 3882804

[B63] Perkell J. Zandipour M. Matthies M. L. Lane H. (2002). Economy of effort in different speaking conditions. I. A preliminary study of intersubject differences and modeling issues. *J. Acoust. Soc. Am.* 112 1627–1641. 10.1121/1.1506369 12398468

[B64] Perkell J. S. Matthies M. L. (1992). Temporal measures of anticipatory labial coarticulation for the vowel/u/- within-subject and cross-subject variability. *J. Acoust. Soc. Am.* 91 2911–2925. 10.1121/1.403778 1629484

[B65] Remijsen B. Gilley L. (2008). Why are three-level vowel length systems rare? Insights from Dinka (Luanyjang dialect). *J. Phonetics* 36 318–344. 10.1016/j.wocn.2007.09.002

[B66] Repp B. H. (2008). Perfect phase correction in synchronization with slow auditory sequences. *J. Mot. Behav.* 40 363–367. 10.3200/JMBR.40.5.363-367 18782711

[B67] Repp B. H. Steinman S. R. (2010). Simultaneous event-based and emergent timing: synchronization, continuation, and phase correction. *J. Mot. Behav.* 42 111–126. 10.1080/00222890903566418 20189907

[B68] Roberts S. (1981). Isolation of an internal clock. *J. Exp. Psychol. Anim. Behav. Process.* 7 242–268. 10.1037//0097-7403.7.3.242 7252428

[B69] Rosenbaum D. A. Patashnik O. (1980). “A mental clock setting process revealed by reaction times,” in *Tutorials in Motor Behavior*, eds Stelmach G. E. Requin J., (Amsterdam: North-Holland Publishing Company), 487–499. 10.1016/s0166-4115(08)61964-0

[B70] Saltzman E. (1995). “Dynamics and coordinate systems in skilled sensorimotor activity,” in *Mind as motion: Explorations in the Dynamics of Cognition*, eds Port R. F. Gelder T. V., (Cambridge, MA: The MIT Press), 149–173.

[B71] Saltzman E. L. Munhall K. G. (1989). A dynamical approach to gestural patterning in speech production. *Ecol. Psychol.* 1 333–382. 10.1207/s15326969eco0104_2

[B72] Saltzman E. Nam H. Krivokapić J. Goldstein L. (2008). “A task-dynamic toolkit for modeling the effects of prosodic structure on articulation,” in *Proceedings of the Speech Prosody 2008 Conference*, eds Barbosa P. A. Madureira S. Reis C., (Campinas: International Speech Communications Association), 175–184.

[B73] Scholz J. P. Schöner G. (1999). The uncontrolled manifold concept: identifying control variables for a functional task. *Exp. Brain Res.* 126 289–306. 10.1007/s002210050738 10382616

[B74] Schöner G. (2002). Timing, clocks, and dynamical systems. *Brain Cogn.* 48 31–51. 10.1006/brcg.2001.1302 11812031

[B75] Scott S. H. (2004). Optimal feedback control and the neural basis of volitional motor control. *Nat. Rev. Neurosci.* 5 534–546. 10.1038/nrn1427 15208695

[B76] Semjen A. (1992). “Determinants of timing in serial movements,” in *Time, Action and Cognition*, Vol. 66 eds Macar F. Pouthas V. Friedman W. J., (Dordrecht: Springer).

[B77] Shaffer L. H. (1982). Rhythm and timing in skill. *Psychol. Rev.* 89 109–122. 10.1037//0033-295x.89.2.109 7089124

[B78] Shaiman S. (2001). Kinematics of compensatory vowel shortening: the effect of speaking rate and coda composition on intra- and inter-articulatory timing. *J. Phonetics* 29 89–107. 10.1006/jpho.2001.0133

[B79] Shaiman S. (2002). Articulatory control of vowel length for contiguous jaw cycles: the effects of speaking rate and phonetic context. *J. Speech Lang. Hear. Res.* 45 663–675. 10.1044/1092-4388(2002/053) 12199397

[B80] Shaiman S. Adams S. G. Kimelman M. D. Z. (1995). Timing relationships of the upper lip and jaw across changes in speaking rate. *J. Phonetics* 23 119–128. 10.1016/s0095-4470(95)80036-0 1787240

[B81] Shouval H. Z. Hussain Shuler M. G. Agarwal A. Gavornik J. P. (2014). What does scalar timing tell us about neural dynamics? *Front. Hum. Neurosci.* 8:438. 10.3389/fnhum.2014.00438 24994976 PMC4063330

[B82] Šimko J. (2009). *The Embodied Modelling of Gestural Sequencing in Speech.* Ph.D. Dissertation, University College Dublin, Dublin.

[B83] Šimko J. Cummins F. (2010). embodied task dynamics. *Psychol. Rev.* 117 1229–1246. 10.1037/a0020490 21038977

[B84] Šimko J. Cummins F. (2011). Sequencing and optimization within an embodied Task Dynamic model. *Cogn. Sci.* 35 527–562. 10.1037/a0020490 21038977

[B85] Šimko J. O’Dell M. Vainio M. (2014). Emergent consonantal quantity contrast and context-dependence of gestural phasing. *J. Phonetics* 44 130–151. 10.1016/j.wocn.2013.11.006

[B86] Sorensen T. Gafos A. (2016). The gesture as an autonomous nonlinear dynamical system. *Ecol. Psychol.* 28 188–215. 10.1080/10407413.2016.1230368

[B87] Spencer R. M. C. Zelaznik H. N. Diedrichsen J. Ivry R. B. (2003). Disrupted timing of discontinuous but not continuous movements by cerebellar lesions. *Science* 300 1437–1439. 10.1126/science.1083661 12775842

[B88] Stevens K. N. (2002). Toward a model for lexical access based on acoustic landmarks and distinctive features. *J. Acoust. Soc. Am.* 111 1873–1891. 12002871 10.1121/1.1458026

[B89] Studenka B. E. Zelaznik H. N. Balasubramaniam R. (2013). The distinction between tapping and circle drawing with and without tactile feedback: an examination of the sources of timing variance. *Q. J. Exp. Psychol.* 65 1086–1100. 10.1080/17470218.2011.640404 22332846

[B90] Tanaka H. Krakauer J. W. Qian N. (2006). An optimization principle for determining movement duration. *J. Neurophysiol.* 95 3875–3886. 10.1152/jn.00751.2005 16571740

[B91] Tilsen S. (2013). A dynamical model of hierarchical selection and coordination in speech planning. *PLoS One* 8:e62800. 10.1371/journal.pone.0062800 23638147 PMC3634742

[B92] Tilsen S. (2016). Selection and coordination: the articulatory basis for the emergence of phonological structure. *J. Phonetics* 55 53–77. 10.1016/j.wocn.2015.11.005

[B93] Tilsen S. (2018). “Three mechanisms for modeling articulation: selection, coordination, and intention,” *Paper Presented in Cornell Working Papers in Phonetics and Phonology*, New York, NY.

[B94] Todorov E. Jordan M. I. (2002). Optimal feedback control as a theory of motor coordination. *Nature Neuroscience* 5 1226–1235. 10.1038/nn963 12404008

[B95] Todorov E. Jordan M. I. (2003). “A minimal intervention principle for coordinated movement,” in *Proceeding of the NIPS’02 Proceedings of the 15th International Conference on Neural Information Processing Systems*, Cambridge, MA: MIT Press, 27–34.

[B96] Trouvain J. (1999). *Phonological Aspects of Reading Rate Strategies.* Saarbrücken: University of the Saarland, 15–35.

[B97] Turk A. E. Shattuck-Hufnagel S. (2007). Multiple targets of phrase-final lengthening in American English words. *J. Phonetics* 35 445–472. 10.1016/j.wocn.2006.12.001

[B98] Turk A. Shattuck-Hufnagel S. (2020). *Speech Timing: Implications for Theories of Phonology, Phonetics, and Speech Motor Control.* Oxford: Oxford University Press. (in press).

[B99] Wilhelms-Tricarico R. (2015). A multi-language speech synthesizer based on syllables as the functional units of speech. *J. Phonetic Soc. Japan* 19 86–99.

[B100] Windmann A. (2016). *Optimization-Based Modeling of Suprasegmental Speech Timing.* Ph.D. Dissertation, Universität Bielefeld, Bielefeld.

[B101] Windmann A. Juraj Š. Wagner P. (2015). “Polysyllabic shortening and word-final lengthening in English,” in *Proceedings of Interspeech 2015*, Dresden, 36–40.

[B102] Winter D. A. (1984). Kinematic and kinetic patterns in human gait: variability and compensating effects. *Hum. Mov. Sci.* 3 51–76. 10.1016/0167-9457(84)90005-8

[B103] Zelaznik H. N. Rosenbaum D. A. (2010). Timing processes are correlated when tasks share a salient event. *J. Exp. Psychol. Hum. Percept. Perform.* 36 1565–1575. 10.1037/a0020380 20731516

